# Redefining the extinct orders Miomoptera and Hypoperlida as stem acercarian insects

**DOI:** 10.1186/s12862-017-1039-3

**Published:** 2017-08-25

**Authors:** Jakub Prokop, Martina Pecharová, Romain Garrouste, Robert Beattie, Ioana C. Chintauan-Marquier, André Nel

**Affiliations:** 1 Department of Zoology, Faculty of Science, Charles University, Viničná 7, 128 43 Praha 2, Czech Republic; 2 Institut de Systématique, Évolution, Biodiversité, ISYEB - UMR 7205 – CNRS, MNHN, UPMC, EPHE, Muséum national d’Histoire naturelle, Sorbonne Universités, 57 rue Cuvier, CP 50, Entomologie, F-75005 Paris, France; 3 The Australian Museum, 1 William St, Sydney, New South Wales 2010 Australia

**Keywords:** Insecta, Acercaria, Late Palaeozoic, Mesozoic, Evolutionary history, Wing venation

## Abstract

**Background:**

The systematic positions of the extinct insect orders Hypoperlida, Miomoptera and Permopsocida were enigmatic and unstable for nearly a century. The recent studies based on new material, especially from the Cenomanian Burmese amber, shed light on evolutionary history of Acercaria resolving Permopsocida as the stem group of Condylognatha. However, the knowledge of the remaining two orders differs significantly.

**Results:**

In this study, we describe new specimens and evaluate morphology of various structures with emphasis on the mouthparts and wing venation. Our results are primary based on revisions of the type specimens with a proper delimitation of taxa Hypoperlida and Miomoptera followed by their significance for the evolutionary history of Acercaria. Three new genera as *Belmomantis* gen. nov., *Elmomantis* gen. nov., and *Mazonopsocus* gen. nov. are designated as members of Palaeomanteidae. The Pennsylvanian *Mazonopsocus* provides a minimum age for calibration, in accordance to the presence of crown acercarians during the late Carboniferous.

**Conclusions:**

This contribution demonstrates that Hypoperlida and Miomoptera are stem groups of Acercaria. The putative clade (Hypoperlida + Miomoptera) is appearing as potential sister group of (Psocodea + (Permopsocida + (Thripida + Hemiptera))).

**Electronic supplementary material:**

The online version of this article (doi:10.1186/s12862-017-1039-3) contains supplementary material, which is available to authorized users.

## Background

The Hypoperlida are an extinct order proposed by Martynov [1] for several enigmatic Permian insects. Since this date several authors placed many Paleozoic taxa in this order, on the basis of wing venation and mouthpart structures, even if the type genus and species *Hypoperla elegans* Martynov, 1928 [1] is based on isolated wings. The Hypoperlida are currently considered as a crucial order that would link the palaeopteran group Palaeodictyopterida with the neopteran clade Acercaria ([2]), under a general scheme of classification that refutes the division of pterygote insects into Palaeoptera and Neoptera, preferring a subdivision into Scarabaeones and Gryllones. Huang et al. [3]) made a revision of the acercarian order Permopsocida, as sister group of the clade (Thripida + Hemiptera). These results were confirmed by Yoshizawa & Lienhardt [4] who used different set of characters like wing base articulations. In the same paper of Huang et al. [3], the type family Hypoperlidae of the Hypoperlida was also revised. The wing venation of *Hypoperla* and related genera showed the acercarian synapomorphies as defined by Nel et al. [5]. Thus the family Hypoperlidae was falling as sister group of all other acercarian insects (Psocodea + (Permopsocida + (Thripida + Hemiptera))), even if they have retained the plesiomorphic condition of the presence of one-segmented cerci, unlike the orders of the crown group Acercaria. Thus the order ‘Hypoperlida’ has to be considered as belonging to the stem group of Acercaria. It remains that ca. fourteen fossil families are currently considered in Hypoperlida. Their positions need to be reconsidered.

The Miomoptera were another extinct order lacking clear synapomorphy to define it [5]. We had recently the opportunity to restudy the type species of the order Miomoptera, and describe new Carboniferous and Permian miomopterans from Mazon Creek (USA), Elmo (USA), and Belmont (Australia). These fossils show wing venation patterns typical of the Acercaria and few differences with the Hypoperlidae. Therefore a revision of both Miomoptera and Hypoperlida is necessary.

## Results

Superorder Clareocercaria (= Acercaria Börner, 1904 [6] sensu lato) (as pan group).

Etymology. Named after Clareo and cercaria pointing out that the presence or absence of cerci play important role in evolution.

Included orders. Miomoptera Martynov, 1927 [7] sensu nov.; Hypoperlida Martynov, 1928 [1] sensu nov. (as stem groups); Acercaria sensu stricto (crown group), comprising Psocodea Hagen, 1865 [8], Permopsocida Tillyard, 1926 [9] (*sensu* Huang et al., 2016 [3]), Thripida Fallen, 1814 [10], Hemiptera Linné, 1758 [11].

**Fig. 1:**
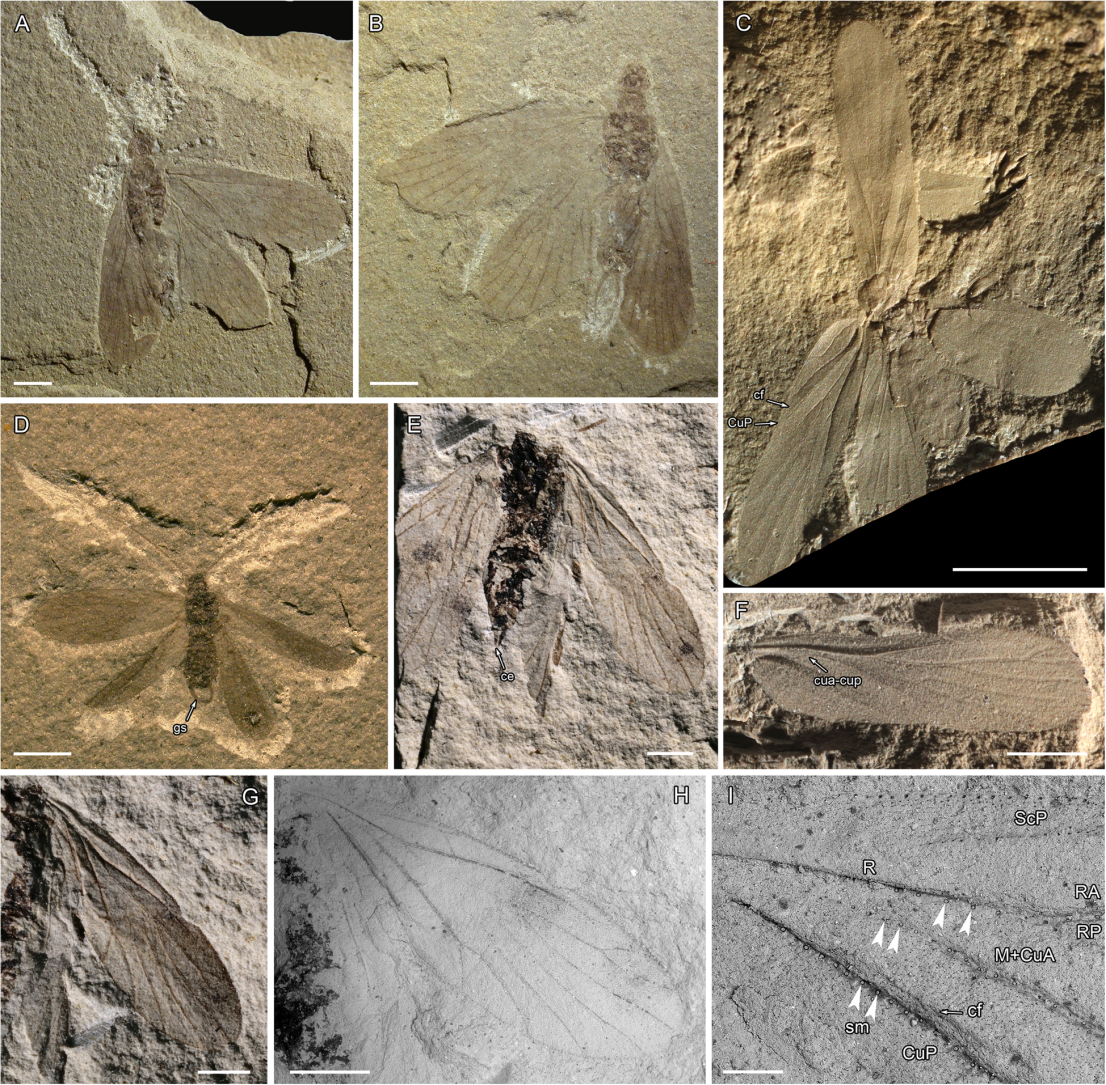
Miomoptera including problematic taxa previously assigned. **a**, **b**, *Palaeomantis aestiva* (Novokshonov, 2000), photograph of holotype PIN No. 1700–4093 (Early Permian, Tshekarda, Russia); **c**, *Perunopterum peruni* Kukalová, 1963, photograph of holotype UK No. 140/1962 (Early Permian, Obora, Czech Republic); **d**, *Delopterum rasnitsyni* Novokshonov, 2000, photograph of holotype PIN No. 1700–4094 (Early Permian, Tshekarda, Russia); (**e**, **g-i**) *Delopsocus latus* (Sellards, 1909) photographs and microphotographs of holotype YPM IP 005384 (Early Permian, Elmo, U.S.A.); (**f**) *Perunopterum peruni* Kukalová, 1963, photograph of specimen UK No. 116/1962 (Early Permian, Obora, Czech Republic), (scale bars represent a**-**d = 2 mm; e-h = 1 mm; *i* = 200 μm). Abbreviations: ce – cerci, cf – claval furrow, gs – gonostyli, sm – sockets of macrotrichia

**Fig. 2:**
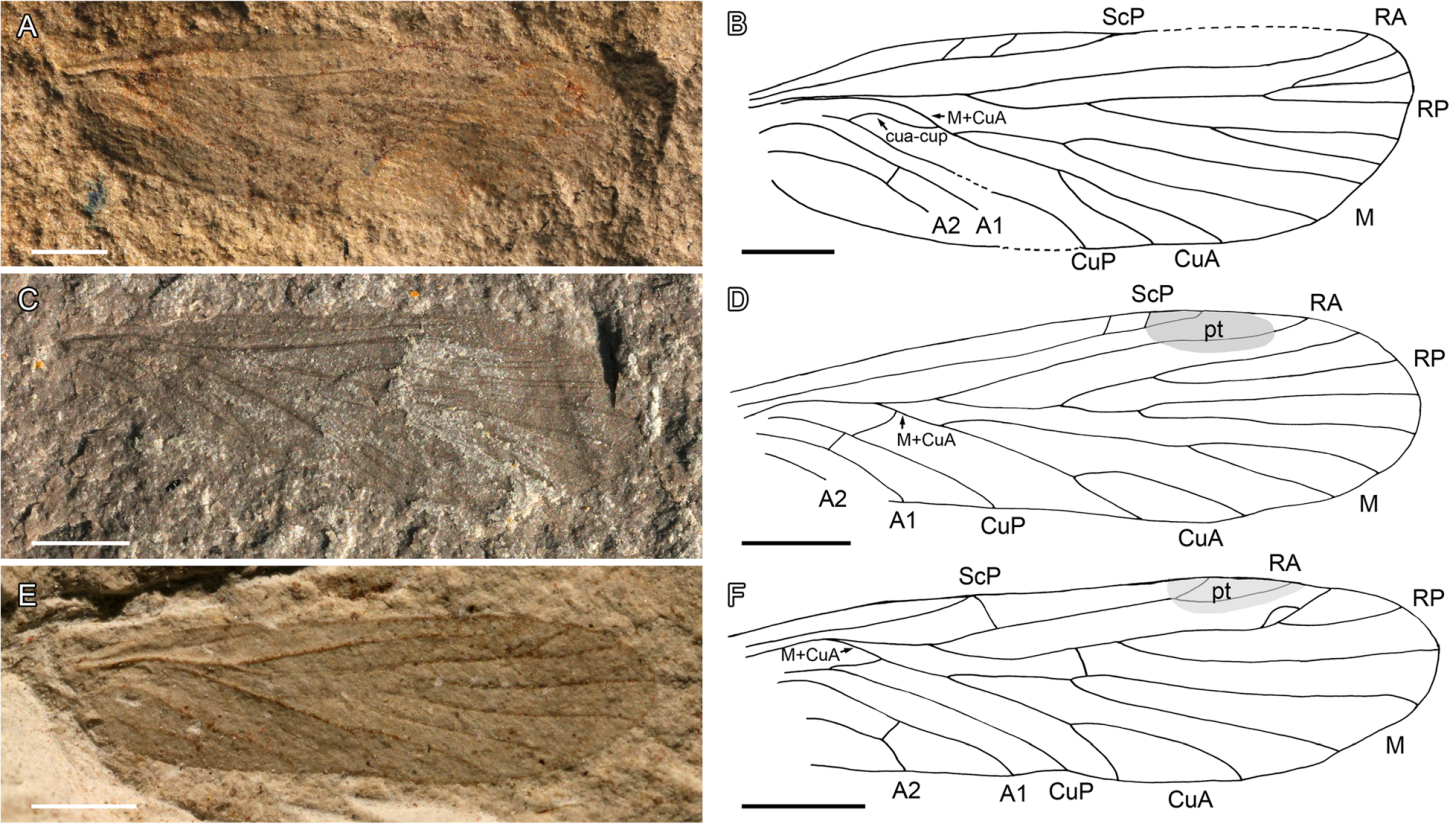
Wing venation in Palaeomanteidae (Miomoptera). **a-b**, *Palaeomantis schmidti* Handlirsch, 1904 (Middle Permian, Tikhie Gory, Russia), photograph and line drawing of specimen PIN No. 5321, forewing reconstruction; (**c-d**) *Belmomantis azari* gen. et sp. nov. (Late Permian, Warners Bay – Belmont area near Newcastle, New South Wales, Australia), holotype AM F.142068, photograph and drawing of forewing; (**e-f**) *Elmomantis engeli* gen. et sp. nov. (Early Permian, Elmo, Kansas, USA), holotype USNM without number, photograph and drawing of forewing (scale bars represent 1 mm)

**Fig. 3:**
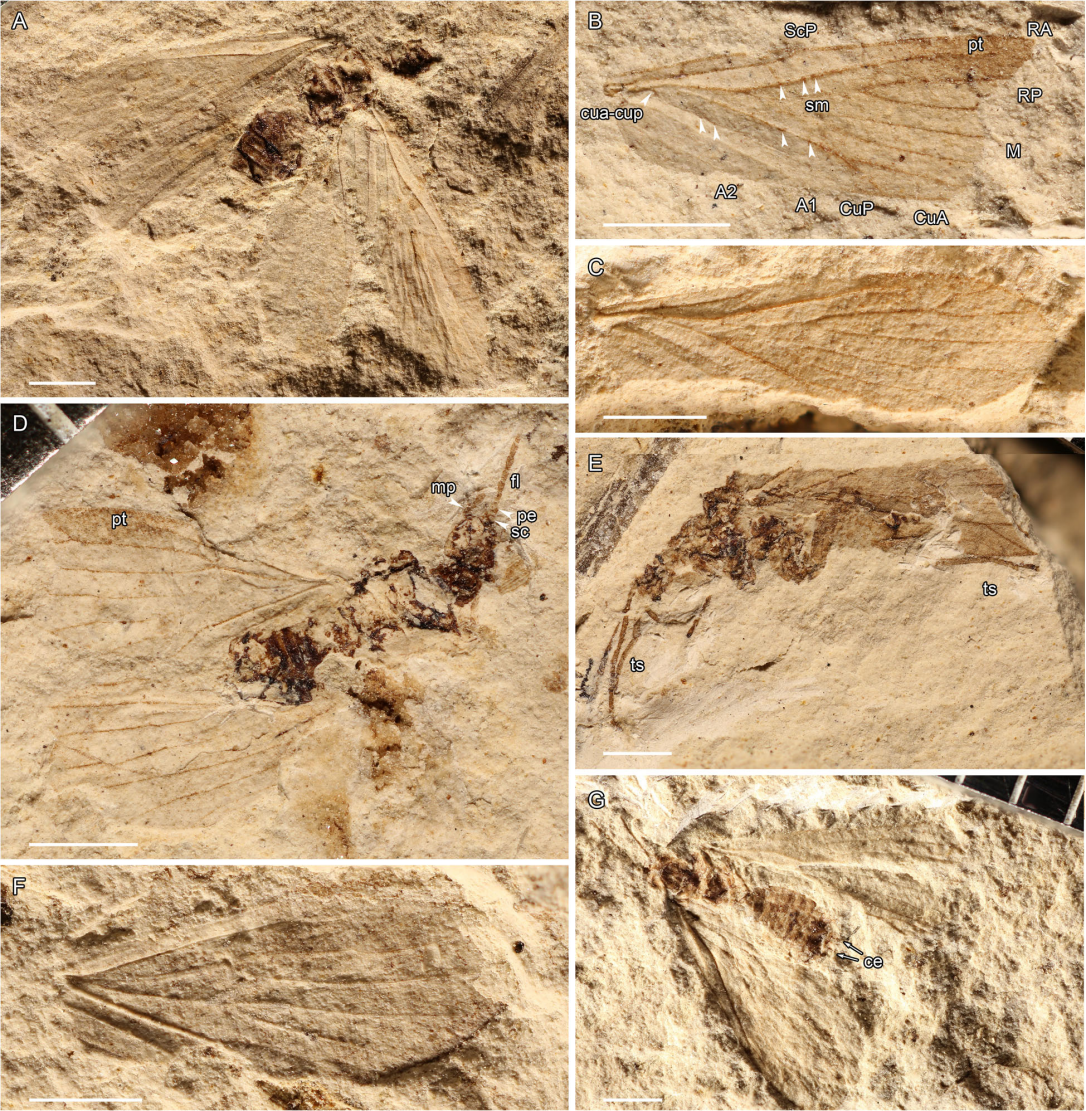
*Delopterum minutum* Sellards, 1909 (Miomoptera), Early Permian, Elmo, Kansas, USA. **a**, photograph of neotype No. MCZ 3295b; (**b**) photograph of forewing venation No. MCZ 3203b; (**c**) photograph of forewing venation No. MCZ 3206; (**d**) photograph of No. MCZ 3209b; (**e**) photograph of No. MCZ 3201a; (**f**) photograph of forewing venation specimen No. MCZ 3296; (**g**) photograph of specimen No. MCZ 13311 (scale bars represent 1 mm). Abbreviations: ce – cerci, fl – flagellum, mp – maxillary palpus, pe – pedicelus, pt – pterostigma, sc – scapus, sm – sockets of macrotrichia, ts – tarsi

Diagnosis. Huang et al. [3] listed a series of body and wing venation synapomorphies for the crown group of Acercaria. Only the following wing venation synapomorphies are present in Hypoperlidae and Miomoptera too: a common stem R + M + CuA, M + CuA separating from R distally; convex CuA immediately emerging from M + CuA (three characters also present in the Archaeorthoptera sensu Béthoux and Nel [12]; long crossvein cua-cup between concave CuP and CuA concave near CuP and convex near CuA, CuA, with its part close to CuP concave and its part close to CuA convex [5]; generally CuA with two branches forming an areola postica, reversed in Thripida and some families of Psocodea and Hemiptera (e.g., Figs. 1e, h, 2b, d, f, 3b, 5a-f).

Remarks. – Acercaria Börner, 1904 currently comprises the family Hypoperlidae, and the orders Psocodea (including ‘Psocoptera’ and Phthiraptera), Thripida (including Thysanoptera) and Hemiptera (Huang et al., 2016 [3]). The name Acercaria means ‘no cerci’, but the Hypoperlidae have one-segmented, reduced cerci, while the Miomoptera seem to have segmented cerci: *Palaeomantis aestiva* Novokshonov, 2000 has long cerci [13] (Rasnitsyn [14] put this species in the genus *Permonikia* without discussion and formal transfert, and *Delopterum rasnitsyni* Novokshonov, 2000 has short cerci [13], but surprisingly long gonostyli (see Figs 1a-b, 1d). Carpenter [15: pl. 2, Fig. 6] also demonstrated copulatory hooks as gonostyli in male of *Dichentomum tinctum* Tillyard, 1926. Nervertheless we prefer to use the name Acercaria here rather than Paraneoptera that has a different meaning, as this group originally comprises the order Zoraptera too. The Zoraptera has been considered as sister group of Acercaria and both taxa have been classified together as Paraneoptera [16, 17]. However, polyneopteran affinities of Zoraptera recently gained further support [18, 19], so that Paraneoptera either has to be rejected as polyphyletic [19] or considered as synonymous with Acercaria [20]. To avoid confusion, we prefer to use here the name Acercaria for the whole clade.

- The Archaeorthoptera is the unique other clade having a common stem M + CuA (more or less connected to R) [12]. But, Archaeorthoptera have CuA with a higher number of distal branches and a concave anterior branch of CuP ending on convex CuA instead of a cua-cup.

- Huang et al. [3] and Yoshizawa and Lienhard [4] proposed in parallel two phylogenetic analyses of the Acercaria. Both demonstrated that the Permopsocida are the sister group of the (Thripida + Hemiptera), interestingly on the basis of two different sets of characters (wing base sclerites in the case of Yoshizawa and Lienhard, head structures in the case of Huang et al. [3].

Order Miomoptera Martynov, 1927 sensu nov. [7]

Type family Palaeomanteidae Handlirsch, 1906 [21]. The other families currently considered as Miomoptera are here excluded from this order.

Type genus and species. *Palaeomantis schmidti* Handlirsch, 1904 [22].

Age range. Late Carboniferous to Middle Permian.

Remarks. The Miomoptera appear as a set of taxa currently supported by no clear synapomorphy. The presence of long cerci in the Palaeomanteidae suggests that it is in more ‘basal’ position than the Hypoperlida (Hypoperlidae) that have very short one-segmented cerci.

The Miomoptera have also been named Palaeomanteida Handlirsch, 1906 (see Zhuzhgova et al. [23]). The Palaeomanteidae and the Hypoperlidae share two synapomorphies, viz. presences in the fore- and hindwings of darkened pterostigmata covering the area between C and anterior branch of RP (including the apex of RA) (see discussion below).

### Palaeomanteidae are stem acercarians

The structure of the basal parts of the median and cubital veins is rather poorly known in the Palaeomanteidae, even if in general, the median vein is considered to be basally fused with radius (or very strongly approximate). In *Elmomantis* gen. nov., *Perunopterum* Kukalová, 1963 [24], *Permodelopterum* Kukalová, 1963 [24], *Archisialis* Martynov, 1933 [25], *Delopsocus* Tillyard, 1928 [26], *Miomatoneura* Martynov, 1927 [7] (note that Martynov (1927; [7]) gave two names for this genus: *Minomatoneura* and *Miomatoneura*) [27], *Palaeomantina* Rasnitsyn, 2004 [14], and some *Palaeomantis* species (e.g., the type species *P. schmidti* Handlirsch, 1904 [22], *P. laeta* Novokshonov and Zhuzhgova, 2002 [28]), a vein that corresponds to the crossvein cua-cup of the Acercaria, is clearly present between CuP and M + CuA [24, 25, 26, 28].

More precisely, in *Palaeomantis laeta*, the basal part of Cu is concave together with the vein that Novokshonov and Zhuzhgova [28] considered as CuA. Thus this alleged concave ‘CuA’ cannot be CuA but either a branch of CuP (as in Archaeorthoptera) or a crossvein. In *Palaeomantis schmidti* Handlirsch, 1904 and *Elmomantis engeli* sp. nov., this vein is concave in its part near concave CuP and more convex near the convex M + CuA. This situation is exactly identical to what can be observed in extant Acercaria [5].

Previous authors that studied the Palaeomanteidae did not consider the relative convexity vs. concavity of the veins to homologize them, but only their relative positions. The situation is the same for the numerous taxa that are currently included in the Hypoperlida (see below the discussion on these taxa).

Unfortunately this vein between CuP and M + CuA seems to be not preserved in *Palaeomantis sylvensis* Martynov, 1940 [29], *P. apicalis* Rasnitsyn, 2004 [14], *P. ostertalis* (Guthörl, 1962) [30], *P. hangardi* (Guthörl, 1962) [30], but the other forewing veins of these taxa correspond to the same pattern present in the other species of *Palaeomantis* [14, 22, 28, 31].

All these fossils have forewing venations typical of Acercaria sensu Huang et al. [3], with the following synapomorphies: a common stem R + M + CuA; a faint crossvein cua-cup; an areola postica; RP and M with few branches; few crossveins. They share with the Hypoperlidae (sensu Huang et al. [3]) the presence of a pterostigmal zone around apex of RA extending below RA, a character absent in all other Acercaria, appearing as a potential synapomorphy of the Hypoperlidae, plus the three-branched RP. They differ from the Hypoperlidae in the median vein separating from CuA very far from their common re-emergence from R + M + CuA. This character is typical to the Palaeomanteidae Handlirsch, 1906 [21], and more precisely to the genera *Palaeomantis* Handlirsch, 1904 [22], *Permodelopterum* Kukalová, 1963 [24], and *Perunopterum* Kukalová, 1963 [24] (Fig 1c, f). They also share the following characters: RP with 3–4 branches, and areola postica elongate.

In conclusion, we consider the Paleomanteidae as representatives of the stem group of the Acercaria, with one important difference with the Hypoperlidae, cerci long, probably two-segmented (Figs 1a-b, 1e); plus a less significant difference, longer stem M + CuA re-emerging from R + M + CuA. Whether Palaeomanteidae and Hypoperlidae are sister groups, as in our phylogenetic analysis (see below), or not is still an opened question because there are not enough characters to decide this important point.

Martynov [7] considered the Palaeomantidae as the type family of the Miomoptera Martynov, 1927 [7]. The Miomoptera have to be considered as belonging to the superorder Acercaria, and should comprise only the family Palaeomanteidae.

The other families currently included in the Miomoptera sensu Carpenter [32], viz. Archaemiopteridae Guthörl, 1939 [33] and Metropatoridae Handlirsch, 1906 [21], are based on incomplete wings (*Miomina* Martins-Neto and Gallego, 1999 [34], *Metropator* Handlirsch, 1906 [21], *Saaromioptera* Guthörl, 1963 [35], *Archaemioptera* Guthörl, 1939 [33]) with crucial structures of wing base not preserved, or wings that have not the acercarian characters (*Tychtodelopterum* Martynova, 1958 [36]) [32, 34, 37], or even as pinnule of fern (*Eodelopterum* Schmidt, 1962 [38]; see Nel et al., 2012 [5]). Thus we exclude them from the Miomoptera and consider them as Insecta: Neoptera of uncertain affinities sit. Nov., after Rasnitsyn [39]. This last author included the families Palaeomantiscidae and Permosialidae in the Miomoptera. After Storozhenko and Novokshonov [40], the Permosialidae have no cua-cup, thus they are probably not related to the Acercaria and to the Palaeomanteidae. The type genus of the Palaeomantiscidae, *Palaeomantisca* Martynov, 1940 [29] was synonymized with the palaeomanteid *Sellardsiopsis* Zalessky, 1939 [39, 41, 42].

Family Palaeomanteidae Handlirsch, 1906 [21].

(= Palaeomantiscidae Rasnitsyn, 1977 [43]).

Type genus and species *Palaeomantis schmidti* Handlirsch, 1904 [22].

Age range: Late Carboniferous to Middle Permian.

New diagnosis. Rasnitsyn (in Rasnitsyn et al. [14]) redefined the family as follows: in forewing, ScP meeting C rather than R; RP with three, rarely two or four pectinate branches; M and CuA with long common stalk and each with two branches (main difference with the Hypoperlidae in which this stalk is quite short); CuP simple, weak (often indistinct); only two (rarely three) anals; crossveins not numerous, weak, often not distinct. We can add to these characters the presence of a vein cua-cup; claval furrow very close to CuP, cerci long, multi-segmented. Rasnitsyn (in Rasnitsyn et al. [14]) also proposed a key to the genera and species of this family.

Other genera accurately attributable to the Paleomanteidae, for the veins CuP, cua-cup, M and CuA visible with their relative convexity: *Belmomantis* gen. nov., *Elmomantis* gen. nov., *Mazonopsocus* gen. nov. *Perunopterum* Kukalová, 1963 [24], *Permodelopterum* Kukalová, 1963 [24] (Dostál [44] provided photographs showing the acercarian pattern of venation and especially the relative convexity of the basal veins of these two latter taxa), *Delopsocus* Tillyard, 1928 [26: Fig. 6].

Some genera are maintained in the Palaeomanteidae for their general wing venations very similar to that of *Palaeomantis*, and with veins CuP, cua-cup, M and CuA visible even if their relative convexities are unknown and should be verified: *Miomatoneura* Martynov, 1927 [7] (but with a 3-branched CuA; *Miomatoneura permica* Kukalová, 1963 seems to have the ‘correct’, acercarian pattern of convexity of the basal veins, after Dostál [44]; *Archisialis* Martynov, 1933 [25]; *Palaeomantina* Rasnitsyn, 1977 [43]; *Sellardsiopsis* Zallesky, 1939 [41] (if the type species of this genus has its cua-cup not preserved, it is the case for *Sellardsiopsis lata* (Martynov, 1940) [29, 42, 43].

Other genera with unknown exact structure of the basal parts of the veins M, CuA, and CuP (in type species): *Balymotikha* Rasnitsyn and Aristov, 2013 [45], *Delopterinus* Rasnitsyn, 2004 [14], *Delopterum* Sellards, 1909 [46], *Miomatoneurella* Martynova, 1958 [36], *Neodelopterum* Rasnitsyn, 2004 [14], *Saaromioptera* Guthörl, 1963 [35], *Stefanomioptera* Guthörl, 1962 [30], *Stigmodelopterum* Rasnitsyn, 2004 [14], *Tridelopterum* Rasnitsyn, 2004 [7, 14, 30, 35, 36, 41, 47]. Thus their attribution to the Acercaria can be based only on the similarities in the distal parts of their wings with the other Palaeomanteidae that clearly have a cua-cup and a fusion of R, M, and CuA with R, with ‘correct’ convexities of the veins.

*Epimastax* Martynov, 1928 [1] (type genus of the Epimastacidae Martynov, 1928 [1]) and *Permonikia* Kukalová, 1963 [24] strongly differs from *Palaeomantis* in the pectinate ScP and RA and the apparent absence of cua-cup [24, 47]. Thus it is not possible to be sure that these taxa belong to the acercarian stem group and to the Palaeomanteidae. Note that *Permonia* Kukalová, 1963 [24] has also pectinate RA and ScP and seems to have a cua-cup. Thus it is probably related to *Epimastax* and all these taxa could belong to the acercarian stem group.

*Urba punctata* Sellards, 1909 [46] is listed among the Palaeomanteidae in the fossilworks internet site. After Sellards [46, p. 169], it is characterized by an anal area with 3–4 veins, which is ‘too much’ for an Acercaria and a Palaeomanteidae.

Rohdendorf [48] did not consider in the Palaeomanteidae the three genera *Delopteriella* Zalessky, 1956, *Miomantisca* Zalessky, 1956, and *Miomatoneurites* Zalessky, 1956, while they were originally put in the Palaeomanteidae [49]. Carpenter [32] synonymized them with *Palaeomantis*. The basal parts of the wings of these taxa are unknown but they all have a long fusion of M with CuA and the veins ScP, RA and RP as in the other Palaeomanteidae [49]. *Miomantisca* has a short ScP, ending on costa at the level of the base of RP, unlike *Palaeomantis schmidti*. These taxa should be revised.

Genus *Palaeomantis* Handlirsch, 1904 [22].

Type species *Palaeomantis schmidti* Handlirsch, 1904 [22].

(Fig. 2a-b).

Material. Handlirsch [22] based his description on the print and couterprint of a forewing overlying a hindwing plus what he considered as a hindwing. Lectotype specimen 5323–5 (182/2), paralectotype specimen 5320–5321. Palaeontological Institute of Russian Academy of Science collection (Moscow, Russia).

Age and outcrop. Middle Permian, Guadalupian, Roadian, lagoonal claystone, Baitugan Formation, Tikhie Gory, Russian Federation.

Redescription. Martynov [7] redescribed this species but he made several errors. Lectotype. A complete isolated forewing, maybe covering a second wing as some veins seem to be double, wing 6.8 mm long, 2.2 mm wide, no trace of coloration preserved, but apparently hyaline; ScP progressively diverging from radius, ending on costa 4.05 mm from wing base, with two oblique crossvein between it and costa; RA with distal fork; RP diverging from RA 2.4 mm from wing base; RP forked 1.3 mm distally, with anterior and posterior branchs forked again 1.9 mm and 1.3 mm respectively distally; a common stem R + M + CuA, with M + CuA separating from R 1.6 mm from wing base; a faint transverse sigmoidal vein cua-cup between CuP and ending on M + CuA far from the base of this vein, with its part close to CuP concave and its part close to CuA convex; relatively neutral M and convex CuA separating 0.9 mm from their common base; M with a deep fork; CuA with a clear areola postica, distinctly longer than high; CuP concave simple; two convex anal veins, first one simple, second with a small crossvein between it and first anal vein.

Paralectotype very similar to the lectotype, thus probably also a forewing, 7.3 mm long, 2.3 mm wide.

*Belmomantis* gen. nov.

Type species. *Belmomantis azari* sp. nov.

Etymology. Named after Belmont, type locality, and *Mantis*, as for many Palaeomanteidae.

Diagnosis. Wing less than 10 mm long; wing length more than two times width; ScP ending near pterostigma, well distal of mid wing level, with several branchlets; RA without branchlets; RP dichotomously four-branched; M + CuA long; cua-cup sigmoidal and ending on M + CuA close to the base of this vein; fork of M well distal of first fork of RP; only two anal veins; very few crossveins.

*Belmomantis azari* sp. nov.

Material. Holotype AM F.142068 (part) and AM F.142069 (c/part), stored in the collection of The Australian Museum, Sydney (New South Wales).

Zoobank LSID urn:lsid:zoobank.org:act:17C7C343-5091-40C9-A287-CE55A93613B9.

Etymology. Named after our friend and colleague Pr. Dany Azar, specialist on fossil insects.

Age and outcrop. Late Permian, mid Lopingian, cca 255 Mya, Newcastle Coal Measures, Warners Bay – Belmont area near Newcastle, New South Wales, Australia [50].

Description. A complete isolated forewing, 6.4 mm long, 1.9 mm wide, no trace of coloration preserved, but apparently hyaline; ScP parallel to radius, ending on costa 4.5 mm from wing base, with two short subapical crossveins between it and costa; RA apparently simple; RP diverging from RA 1.8 mm from wing base; RP forked 1.1 mm distally, with anterior and posterior branches forked again 0.8 mm distally; a common stem R + M + CuA, with M + CuA separating from R 1.2 mm from wing base; a faint transverse vein cua-cup between CuP and M + CuA; M and CuA separating 0.6 mm from their common base; M with a deep fork; CuA with a clear areola postica, longer than high; CuP concave simple; two convex anal veins, both apparently simple.

Discussion. This fossil would fall near the genus *Permonikia*, after the key to species of Rasnitsyn (in [14]) for the following characters: M + CuA long; only two anal veins; wing less than 10 mm long; wing length more than two times width; RP four-branched; ScP with several branchlets. It differs from *P. permoniki* Kukalová, 1963 [24] and *P. aestiva* (Novokshonov, 2000) [13] in the ScP ending near the pterostigma, RA without branchlets, fork of M well distal of first fork of RP, forks of RP dichotomous instead of being pectinate, very few crossveins. This fossil differs from *Palaeomantis* in the ScP ending on costal margin well distal of the mid wing level.

*Elmomantis* gen. nov.

Type species. *Elmomantis engeli* sp. nov.

Etymology. Named after the Elmo site and *Mantis*, as for many Palaeomanteidae.

Diagnosis. ScP touching C and apparently ending on RA distally as a transverse vein between costa and RA; absence of crossveins between main veins; CuA with only one fork; cua-cup sigmoidal and ending on M + CuA far from the base of this vein; RP three-branched; a distinct pterostigma; stem of M long; absence of a crossvein below pterostigma between RA and RP.

*Elmomantis engeli* sp. nov.

Material. Holotype specimen USNM without number, Smithsonian Institution, National Museum of Natural History, Washington, USA.

Zoobank LSDI urn:lsid:zoobank.org:act:73D721C8-2565-4D03-9D8F-7CD14942F838.

Etymology. Named after our friend and colleague Pr. Michael S. Engel, specialist on fossil insects.

Age and outcrop. Elmo Limestone member of the Wellington Formation, Lower Permian, Elmo, Kansas, USA.

Diagnosis. As for the genus.

Description. A nearly complete isolated forewing, with only basal part of anal area not preserved, wing 5.7 mm long, 1.6 mm wide, apparently hyaline but with a darker elliptical pterostigmal zone around apex of RA extending below RA; ScP progressively diverging from radius, touching costa 0.4 mm distal of base of RP, 2.0 mm from wing base, and apparently ending on RA distally as a transverse vein between costa and RA; RA with distal fork; RP diverging from RA 1.6 mm from wing base; RP forked 1.5 mm distally, with anterior branch forked again 1.3 mm distally; a common stem R + M + CuA, with M + CuA separating from R 0.8 mm from wing base; a faint transverse vein cua-cup between CuP and M + CuA, its part near CuP being more concave than its part near CuA; M and CuA separating 0.9 mm from their common base; M + CuA and CuA strongly convex; M neutral, with a deep fork; CuA with a clear areola postica, longer than high; CuP concave simple; two convex anal veins, first one simple, second with a small anterior branch ending on first anal vein.

Discussion. *Elmomantis* is characterized by a combination of characters that are not present in the other palaeomanteid genera, viz. the shape of the ScP touching C and apparently ending on RA distally as a transverse vein between costa and RA, the absence of crossveins between the main veins, CuA with only one fork, RP three-branched, a distinct pterostigma. In the key to Palaeomanteidae of Rasnitsyn (in [14]), *Elmomantis* would fall near *Stigmodelopterum pterostigmalis* Rasnitsyn, 2004 (in [14]), from which it differs in the longer stem of M and absence of a crossvein below pterostigma between RA and RP. Note that the structure of ScP is unknown in *Stigmodelopterum pterostigmalis*.

*Delopsocus* Tillyard, 1928 gen. rest.

Type species. *Delopsocus elongatus* Tillyard, 1928. Other species. *Delopsocus fasciatus* Tillyard, 1928, *Delopsocus furcatulus* (Martynov, 1930), *Delopsocus kamensis* (Martynov, 1938), *Delopsocus kansanum* (Carpenter, 1939), *Delopsocus lepidus* (Kukalová, 1963), *Delopsocus sinuosus* (Kukalová, 1963), *Delopsocus stenopterus* Rasnitsyn, 2004, *Delopsocus latus* (Sellards, 1909).

Remark. Carpenter [32] synonymized the genus *Delopsocus* with *Palaeomantis*. Rasnitsyn (in [14]) considered them as different genera but without formally restoring the genus *Delopsocus*. We restore the genus *Delopsocus* because the type species *D. elongatus* differs from the type species of *Palaeomantis* in two important characters: ScP is emitting a posterior branch (or strong crossvein towards RA at its apex in the former while it is absent in the latter; vein cua-cup is in a very basal position in the former while it is distinctly more distal in the latter. Both these characters are present in the majority of the species currently included in *Delopsocus*. *D. stenopterus* is based on an incomplete wing, with basal structures and apex of ScP missing [14].

*Delopsocus latus* (Sellards, 1909) [originally *Delopterum latum* Sellards, 1909].

Material. Holotype 94 (hindwing) in Sellards’ collection. Redescribed and figured by Tillyard [26: fig. 8], who listed several other specimens. Here we redescribe the specimen YPM 5384A–B (part and counterpart) in Yale Peabody Museum collection.

Age and outcrop. Elmo Limestone member of the Wellington Formation, Lower Permian, Elmo, Kansas, USA.

Description of specimen No. YPM 5384.

Body not well preserved, head only partly visible; apical part of an antenna with ca. five short flagellomeres visible; thorax 1.65 mm long, 1.25 mm wide; legs not visible; abdomen 2.46 mm long, 1.09 mm wide; cerci partially preserved, probably two-segmented, 0.39 mm long.

Forewing 5.65 mm long, 1.82 mm wide; ScP 2.37 mm long, with an apical fork, anterior branch ending on C while posterior one ends on RA; R divided into RA and RP 1.80 mm from wing base; RA simple; RP with three branches; M and CuA basally fused with R, separating again 2.09 mm from wing base in a widened part of R; R + M + CuA, R, RA, and M + CuA distinctly convex; a faint vein cua-cup between concave CuP and point of re-emergence of M + CuA, convex near M + CuA and vanishing near CuP (preservation of absence of connection with CuP as in many Hemiptera?); M and CuA separating 0.78 mm from their connection to R; M with two branches; CuA with two branches (areola postica longer than wide); CuP simple; claval furrow well discernable running close and parallel to CuP (see Fig. 1i); anal area poorly preserved, but with only convex A1 discernable, a crossvein between CuA1 and M2, one between M1 and RP; veins R, RA, M + CuA, and CuP with large insertions of basal sockets of macrotrichia, see Fig. 1i), surface of wings covered with microtrichia. Hindwings partly preserved, of same sizes as forewings; preserved veins identical to those of forewings; a dark zone around apex of RA (either a pterostigma or just dense pattern of microtrichia).

Remark. The redescription of this fossil confirms the attribution of *Delopsocus* to the acercarian stem group (typical wing venation), together with the presence of presumably two-segmented cerci in the Palaeomanteidae. Moreover, the pattern of prominent basal sockets of macrotrichia on main longudinal veins R, and M + CuA (Fig. 1i) also occurs in other members of Acercaria, like a hemipteran *Mundus nodosus* Becker-Migdisova, 1960 and many extant psocopterans, e.g. [51]. Another important point is the presence of claval furrow (cf) medially running closely parallel to CuP. This character is also present in *Perunopterum peruni* Kukalová, 1963 where it is apically diverging from CuP (see Fig. 1c).

*Delopterum* Sellards, 1909.

Type species. *Delopterum minutum* Sellards, 1909. Other species. *Delopterum anale* Martynov, 1928, *Delopterum candidum* Zhuzhgova, 2002, *Delopterum commune* Rasnitsyn, 2004, *Delopterum iljinskiense* Martynova, 1961, *Delopterum incertum* Martynov, 1928, *Delopterum insigne* Martynov, 1928, *Delopterum kaltanicum* Martynova, 1961, *Delopterum latum* Sellards, 1909, *Delopterum pantherinum* Rasnitsyn, 2004, *Delopterum radtshenkoi* Martynova, 1961, *Delopterum rasnitsyni* Novokshonov, 2000, *Delopterum truncatum* Kukalová, 1963, *Delopterum zonatum* Rasnitsyn, 2004. A revision of the different species in this genus will be necessary to verify their value.

*Delopterum minutum* Sellards, 1909.

Remark. The holotype of *D. minutum* No. MCZ 3979 is lost, after Carpenter [52], who designated a neotype the specimen MCZ 3295a, b (Fig. 3a). The original type shows remarkably well preserved cerci [15: pl. 2, fig. 3]. He also studied several other specimens, also from the Museum of Comparative Zoology at Harvard, USA. We restudy some of them of great interest for the wing venation and other body structures of this genus.

Descriptions.

**Specimen 3203a, b** (Fig. 3b). Although this fossil is an incomplete forewing, it shows the acercarian pattern of venation, viz. ScP reaching costal margin in proximal third of wing with one oblique crossvein; CuA is basally fused with M and R in a convex vein; between concave CuP, there is a faint vein cua-cup that ends in M + CuA at its point of separation with R, which is basally concave and distally convex. Otherwise, forewing 1.2 mm wide; R divided into RA and RP 1.1 mm from wing base; R + M + CuA, R, RA, and M + CuA distinctly convex; RA simple; M and CuA separating again 0.5 mm from wing base in a widened part of R; M and CuA separating 0.7 mm from their common base; M with two branches; CuA with two branches (areola postica longer than wide); concave CuP simple; anal area with only A1 and A2, a crossvein between A1 and A2; no visible crossvein between CuA1 and M2 or between M1 and RP; veins R, RA, M + CuA, and CuP with large insertions of macrotrichia; surface of wing covered with microtrichia; a darkened zone (pterostigma?) surrounding apex of RA.

**Fig. 4:**
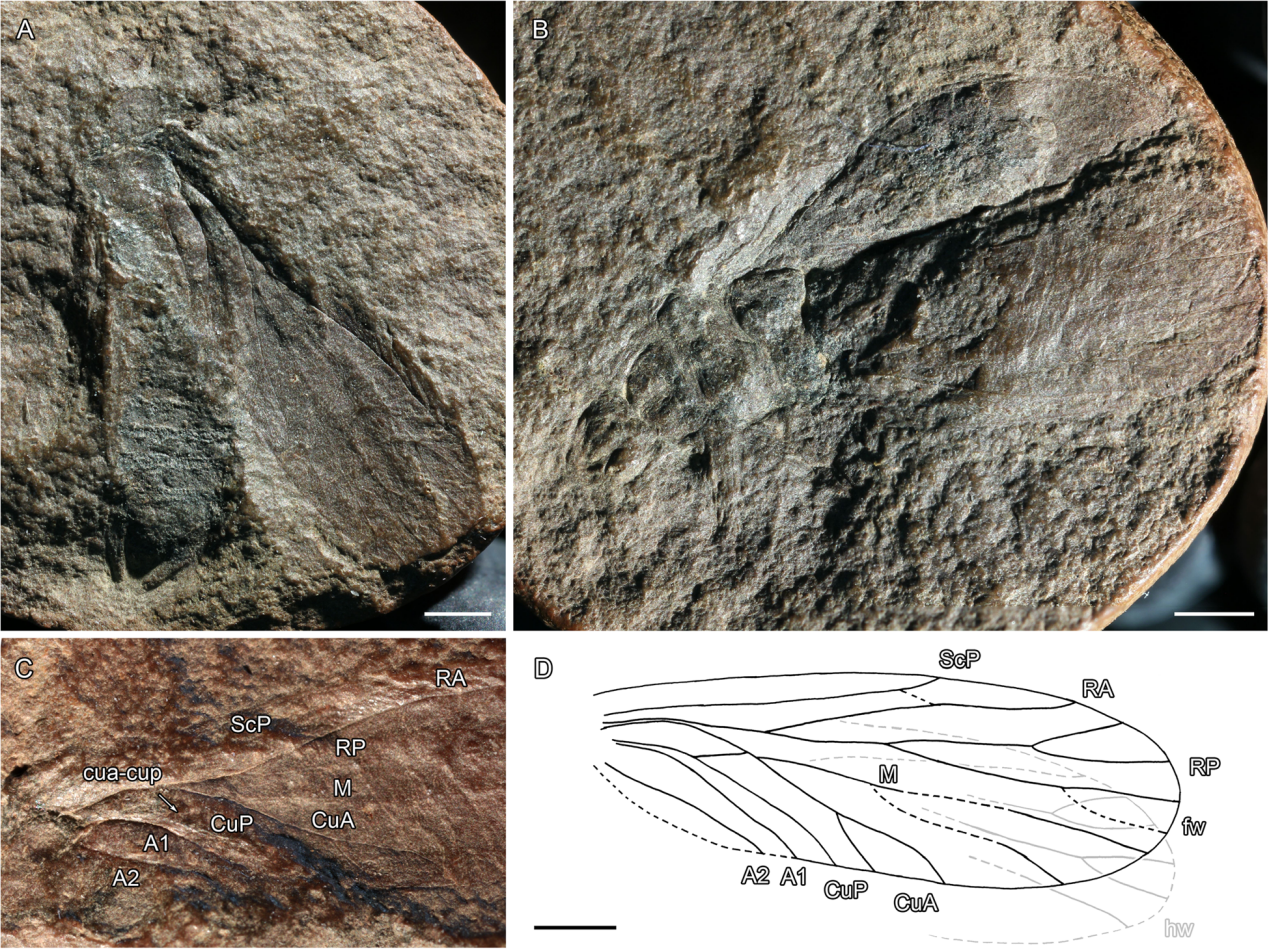
*Mazonopsocus testai* gen. et sp. nov., Paleomanteidae (Miomoptera), Pennsylvanian (Moscovian), Mazon Creek, Illinois, USA). **a** photograph of habitus holotype FM TVT1991a (part); (**b**) photograph of habitus holotype FM TVT1991b (c/part); (**c**) photograph of detail wing base FM TVT1991a (part); (**d**) line drawing of fore- and hindwing venation (scale bars represent 1 mm)

**Specimen 3206** (complete forewing) (Fig. 4c). Wing 4.75 mm long, 1.49 mm wide; ScP 1.70 mm long, with a humeral crossvein perpendicular to it and to C, and a distal fork, anterior branch ending in C and posterior branch ending in RA; RA apparently simple; RP with three branches; M with two branches; areola postica longer than wide; pattern of veins at wing base identical to that of specimen 3203, with a cua-cup basally concave and distally convex, aligned with distal part of M + CuA; a darkened zone (pterostigma?) surrounding apex of RA, poorly visible.

**Specimen 3209** (body with two wings) (Fig. 3d). This specimen is of interest for the very clear pterostigma covering all the area around the apex of RA (apparently simple) and the most anterior branch of RP; general shape of venation identical to that of 3206, but basal part of wing rather poorly preserved, although cua-cup is visible, identical to those of 3206 and 3203. This fossil also shows an antenna with at least six flagellomeres, slightly longer than wide and scape and pedicel broader than flagellomeres; maxillary palp with three visible segments.

**Specimen 3295** (neotype, body with four wings) (Fig. 3a). This specimen shows the hindwings that have the same venation as the forewings, at least in their distal two-thirds. Unfortunately, the wing bases are poorly preserved.

**Specimen 3201a, b** (body with a forewing clearly visible in its distal half) (Fig. 3e). The interest of this fossil is the clearly visible distal fork of RA at the level of pterostigma. This fork is not visible in the previous specimens, probably due to a problem of preservation. The distal two-thirds of the venation is the same as in other specimens. The legs are visible. Carpenter [52] counted four posterior tarsomeres, but he has confused the long tibia with a basal tarsomere (hindleg). There are three basal tarsomeres of the same length, obliquely ‘cut’ at their apices plus two cylindrical apical tarsomeres, narrower than the basal ones, two claws and an arolium between them; one strong seta at apex of posterior tibia. Foreleg with basal long three segments.

**Specimen 3296** (complete wing) (Fig. 3f). This wing also shows a distal fork of RA.

**Specimen 13,311** (body with poorly preserved wings) (Fig. 3g). The interest of this fossil is the presence of the curved cerci, longer than in the Hypoperlidae.

Remark. *Delopterum minutum* shares with *Delopsocus* the very basal position of cua-cup. It differs from *Delopsocus latus* at least in the distally forked RA, larger pterostigma, presence of a basal crossvein perpendicular to C and ScP, absence of a crossvein between RP and M1, and absence of a crossvein between M2 and areola postica.

*Mazonopsocus* gen. nov.

Type species. *Mazonopsocus testai* sp. nov.

Etymology. Composite name after Mazon Creek (type locality) and *Psocus* (genus name).

Diagnosis. Wings elongated, ScP ending on Costa behing midwing, RA simple, RP with three or four branches, M and CuA basally fused with R, distinctly convex M + CuA diverging from R; a faint vein cua-cup between concave CuP and point of re-emergence of M + CuA, convex near M + CuA and concave near CuP; M with two branches; CuA with two branches (areola postica); faint CuP simple, anal area with two convex veins as A1 and A2.

*Mazonopsocus testai* sp. nov.

Material. Holotype FM TVT1991a (part) and FM TVT1991b (c/part), stored in the collection of The Field Museum, Chicago (Illinois, USA), originally from Thomas V. Testa collection.

Zoobank LSDI urn:lsid:zoobank.org:act:683466BB-E44B-4250-B986-4A54456D97D8.

Etymology. Named after the collector Thomas V. Testa who deposited type specimen to The Field Museum in Chicago.

Age and outcrop. Upper Carboniferous, Pennsylvanian, Westphalian C-D (Moscovian), ca. 300 Mya; Mazon Creek, Francis Creek Shale Member, Carbondale Formation, Illinois, USA.

Description. A nearly complete body with a fore- and a hindwing preserved.

Head 1.16 mm long, 1.06 mm wide; mouthparts and antennae not visible; thorax 2.06 mm long, 1.16 mm wide; legs not visible; abdomen 4.9 mm long, 1.82 mm wide; two symmetrical slightly curved appendages emerging well before apex of abdomen (?gonostyli), 1.20 mm long.

Forewing well visible, about 7.3 mm long, 2.52 mm wide; ScP 4.36 mm long, with an apical fork, anterior branch ending on C while posterior one ends on RA; R divided into RA and RP 2.30 mm from wing base; RA simple; RP with preserved three branches; M and CuA basally fused with R, separating again 1.31 mm from wing base; R + M + CuA, R, RA, and M + CuA distinctly convex; a faint vein cua-cup between concave CuP and point of re-emergence of M + CuA, convex near M + CuA and concave near CuP; M and CuA separating 0.95 mm from their common base, close to apex of cua-cup; M with two branches; CuA with two branches (areola postica about as long as wide); CuP simple, apparently basally very close to A1; anal area poorly preserved, but A1 and A2; no visible crossvein between CuA1 and M2, possibly one between M1 and RP. Hindwing only faintly visible below forewing, probably of the same size, RP with both main branches secondarily bifurcated.

Remark. Although the common stem M + CuA is relatively shorter than in some other Palaeomanteidae, the cua-cup is ending into this stem, unlike in the Hypoperlidae in which it ends into CuA or very close to it. Thus this fossil is attributable to the former family. It corresponds to the oldest record of the Miomoptera sensu nov., in the late Carboniferous. Rasnitsyn [39: fig. 214] noted another formerly undescribed specimen from the same locality attributable to Palaeomanteidae with poorly preserved wing bases. Oudard [[53]: figs 14, 17] also figured some forewings from the Gzhelian of Montceau-les-Mines with all the distal structures of venation typical of the Palaeomanteidae, including the long stem M + CuA and the areola postica.

Order Hypoperlida Martynov, 1928 [1] (sensu nov.).

Type family. Hypoperlidae Martynov, 1928 [1]. All the other families currently in the Hypoperlida are here excluded from this order (see below).

Diagnosis. Wing venation very similar to those of the Palaeomanteidae, except mainly in the shorter stem M + CuA. Cerci one-segmented and very short, unlike the long cerci of the Palaeomanteidae.

Family Hypoperlidae Martynov, 1928 [1] (= Kaltanelmoidae Rohdendorf, 1961 [48]).

Type genus. *Hypoperla* Martynov, 1928 [1] (four species: *H. elegans* Martynov, 1928 [1], *H. grata* Novokshonova, 1998 [54], *H. nobilis* Novokshonov, 1995 [55], and *H. vaulevi* Novokshonov, 2001 [56]),

Age range. Permian.

Other genera *Kaltanelmoa* Rohdendorf, 1961 [48] (single species: *K. sibirica* Rohdendorf, 1961 [48]), *Boreopsocus* Shcherbakov, 1994 [57] (two species: *B. danksae* Shcherbakov, 1994 [57] and *B. ficticius* Novokshonov, 1995 [55]), *Onthomastax* Rasnitsyn & Aristov, 2013 [45] (single species: *O. coprinus* Rasnitsyn & Aristov, 2013 [45]), and possibly some of the species previously attributed to the genus *Idelopsocus* Zalessky, 1929 [58], viz. *I. galinae* Novokshonov, 2001 [56], *I. incommendatus* Novokshonov et al., 2002 [59], *I. levis* Novokshonov, 1995 [55], *I. mutovinus* Rasnitsyn and Aristov, 2013 [45], and *I. splendens* (Zalessky, 1948) [60], but maybe not *Idelopsocus diradiatus* Rasnitsyn, 1996 [in 61], *Idelopsocus tartaricus* Zallesky, 1929 [58], and *Idelopsocus arcuatus* (Martynov, 1928) [1] (see below).

Position of the Hypoperlidae in Acercaria. Rasnitsyn [43] included seven genera in Hypoperlidae: *Hypoperla*, *Hypoperlopsis* Zalessky, 1948 [60], *Martynopsocus* Karny, 1930 [62], *Kaltanelmoa* Rohdendorf, 1961 [48], *Fatjanoptera* Martynova, 1961 [63], *Tshunicola* Rasnitsyn, 1977 [43], and *Tshekardobia* Rasnitsyn, 1977 [43]. The latter three genera are discussed below. Shcherbakov [57] restricted the Palaeozoic Hypoperlidae to embrace only the four genera *Hypoperla*, *Idelopsocus* Zalessky, 1929 [58], *Kaltanelmoa*, and *Boreopsocus* Shcherbakov, 1994 [57].

Martynov [1] erected the family Dinopsocidae for the genus *Dinopsocus* Martynov, 1928 [1]. Karny [62] proposed the new names *Martynopsocus* for the genus and Martynopsocidae because of the existence of a psocid genus *Dinopsocus* Banks, 1920. Laurentiaux [64] listed the Martynopsocidae in the Permopsocida. Rasnitsyn [45] synonymized the Martynopsocidae with the Hypoperlidae. The type species is *Martynopsocus arcuatus* (Martynov, 1928) [1]. Martynov [1] suggested that *Idelopsocus* and *Dinopsocus* could be the same genus. Laurentiaux [63] included the genus *Idelopsocus* (type species *I*. *tataricus* Zallesky, 1929 [58]) in the Martynopsocidae. Carpenter [32] proposed the generic synonymy of *Idelopsocus* Zalessky, 1929 [58] with *Martynopsocus*, but did not take into account the date priority of *Idelopsocus* on *Martynopsocus*. Therefore the valid genus name is *Idelopsocus*.

**Fig. 5:**
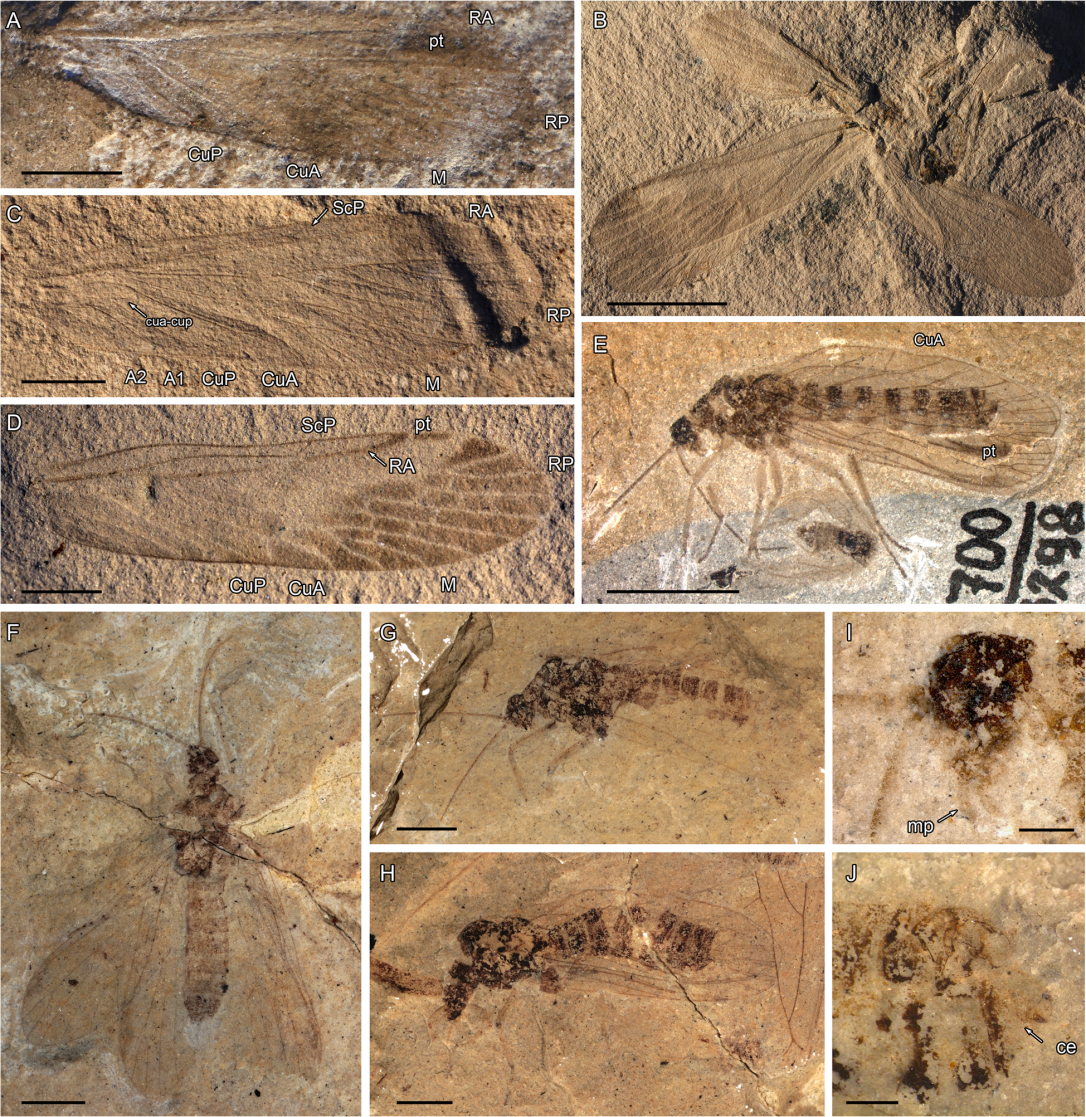
Hypoperlida: (**a-d**), *Hypoperla elegans* Martynov, 1928: (**a**) Holotype PIN 2050/17 (Late Permian, Soyana River, Archangelsk Region, Russia), photograph of forewing venation (scale bar represents 2 mm); (**b**), PIN No. 3353/415 (Late Permian, Soyana River, Archangelsk Region, Russia), photograph of body with fore- and hindwings venation, specimen. (**c**), PIN No. 117/968 (Late Permian, Soyana River, Archangelsk Region, Russia), photograph of forewing, specimen; (**d**), PIN No. 3353/471 (Late Permian, Soyana River, Archangelsk Region, Russia), photograph of hindwing, specimen. **e-j** ‘*Idelopsocus*’ sp., (**e**) ‘*Idelopsocus*’ cf. *splendens*, PIN 1700/3298, photograph of habitus; (**f**) *Idelopsocus* sp., PIN 1700–3257 (Early Permian, Tshekarda, Russia), habitus in dorsal view; (**g**) *Idelopsocus* sp., PIN 124–118 (Early Permian, Tshekarda, Russia), habitus in lateral view; (**h**) *Idelopsocus* sp., PIN 1700/479 (Early Permian, Tshekarda, Russia), habitus in dorsolateral view; (**i**) PIN 1700/3298 (Early Permian, Tshekarda, Russia) lateral view of head with maxillary palps; (**j**) ‘*Idelopsocus’ splendens* PU 2/129 (Early Permian, Tshekarda, Russia), apex of abdomen with one segmented cerci (scale bars represent a, c, d, f-h = 2 mm, b, e = 5 mm, i, j = 0,5 mm). Abbreviations: ce – cerci, mp – maxillary palps, pt – pterostigma

The venation of *Hypoperla elegans* (type species of Hypoperlidae, type family of the order Hypoperlida) is typical for Acercaria by having a common stem R + M + CuA; M + CuA separating from R distally; convex CuA immediately emerging from M + CuA; long crossvein cua-cup between concave CuP and CuA, which is concave near CuP and convex near CuA; CuA with an areola postica (see Fig. 5c-d). Nevertheless, *Hypoperla elegans* differs from the Permopsocida in several important plesiomorphies: RP with a series of parallel posterior branches instead of a single fork, as in modern Acercaria and Permopsocida (a likely plesiomorphy because numerous posterior branches of RP are known in the ground plans of polyneopterous orders and in Neuropterida and Panorpida); no distinct angle of radius at base of M + CuA; pterostigma more ‘rudimentary’ and consisting of a darker zone covering apical parts of ScP, RA, and apical part of area between RA and RP, not delimited posteriorly by RA. The same pattern of venation occurs in *Hypoperla grata* and *Hypoperla vaulevi*.

The venation of *Idelopsocus tataricus* is clearly of acercarian type, showing a convex CuA emerging with concave M from a common stem with R, a long brace cua-cup between concave CuP and CuA, concave near CuP and convex near CuA, and two convex simple anal veins. The CuA of *I. tataricus* is simple, concave ScP ends on RA, and concave RP and M both have three branches with few crossveins [58]. This venation is closer to modern Acercaria than to that of *Hypoperla*. It differs from the Permopsocida in lacking a strong angle between RA and basal stem R + M + CuA, and not having a sclerotized pterostigma.

The venations of *I. arcuatus* strongly resembles that of *I. tataricus* from which it differs in the presence of an areola postica (CuA forked) [1].

*Idelopsocus diradiatus* also has a venation closer to non-hypoperlidan Acercaria in that the RP only has two branches, and M with only three branches, but lacking any angle in the course of R at the base of M + CuA [65]. *Idelopsocus diradiatus* has a forked CuA, unlike *I. tataricus*. *Idelopsocus tataricus* and *I. incommendatus* share similar venation characters except for presence of an areola postica [66]. The venation appears somewhat variable among the species currently placed in *Idelopsocus*, especially in the number of main vein branches. Unlike *Hypoperla*, where only the distal parts of the wings have darkened membranes, species of *Idelopsocus* possess sclerotized pterostigmata in fore- and hind wings (Fig. 5e-f) [1], not homologous to that of Permopsocida because the pterostigmata cover a zone crossing the distal area between the anterior wing margin and RA and part of the area between RA and RP. In Permopsocida, the pterostigmata are delimited posteriorly by RA. *Idelopsocus mutovinus* is probably also a Hypoperlidae, although the basal part of the vein CuA is not clearly discernable [45]. *Idelopsocus diradiatus* and *Idelopsocus splendens* have five-segmented tarsi (specimens PIN 1700/3298 or PU 2/129 attributed to *I. splendens* by Novoskshonov [56] and Rasnitsyn [2]); while the type specimen of *I. splendens* is an isolated wing originally described as *Hypoperlopsis splendens*] [60]. This tarsal formula is a plesiomorphy in Acercaria and most insects.

*Boreopsocus* has a venation most suggestive to that of Permopsocida, with RP having a distal fork, pterostigmata in fore- and hind wings delimited by a posterior curve of RA, with a crossvein below it and RP (but narrower than in Permopsocida, except *Stenopsocidium*). Unlike Permopsocida [23, 57], it lacks an angular R, and possesses five-segmented tarsi. *Kaltanelmoa sibirica* (based on the basal two-thirds of an isolated wing) also has a venation typical of Acercaria (courses of M and cubital veins, simple fork of CuA). RP and M in this species appear to be simply forked, as in modern acercarians and Permopsocida, but R lacks an angle in its course distal to base of M. The area of the putative pterostigma is hardly preserved [48].

In summary, the family Hypoperlidae *sensu* Shcherbakov [57] appears to be a ‘group’ of acercarian genera, but they lack a clear apomorphy that could support them as a clade. They may represent a paraphyletic ‘evolutionary grade’ (with regard to wing venation and number of tarsomeres) from *Hypoperla* to *Boreoposocus*, the latter is sharing several apomorphies with Permopsocida (similar pterostigmata and venation). The venations of the *Idelopsocus* species could represent ‘intermediate’ stages, having reduced branchings in RP and M, compared to the situation observed in *Hypoperla*, but with a particular pterostigma different from *Boreopsocus* and Permopsocida. Interestingly, a strikingly similar phenomenon happened during the evolution of the odonatopteran pterostigmata: the basal clades (Geroptera Brodsky, 1994 [67], Meganisoptera Martynov, 1932 [68]) have no pterostigma, whereas Odonata have a pterostigma delimited posteriorly by RA [69]. The pterostigma in the ‘intermediate’ clade Protanisoptera Carpenter, 1931 [70] is almost identical in shape and position to that of *Idelopsocus* [71].

### Phylogenetic analysis of the Acercaria sensu lato

**Fig. 6:**
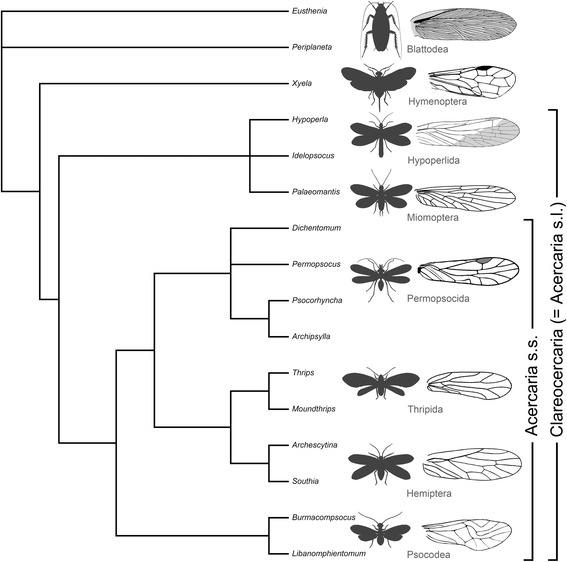
Strict consensus cladogram (MP). Small icons indicate habitus for each order and the corresponding type of forewing venation

Huang et al. [3] proposed a phylogenetic analysis of the Acercaria sensu stricto in which the Hypoperlidae falls as sister group of the crown group. We use the same set of 63 characters as in Huang et al. [3] supplementing the set of taxa by a representative of the Miomoptera (*Palaeomantis aestiva*) (see matrix of characters in Additional files 1, 2 and 3). Using maximum parsimony (MP), we have obtained nine equally most parsimonious cladograms, length = 90 steps; consistency index CI = 0.8111; CI excluding uninformative characters = 0.8023; RI = 0.8859; RC = 0.7186. The Acercaria sensu lato are monophyletic, supported by the wing venation characters only, and appear as sister group of the Holometabola (Fig. 6). The hierarchy of the crown group Acercaria sensu stricto is the same as in Huang et al. [3], supported by the same set of synapomorphies. The two groups Hypoperlidae and Miomoptera fall together in the same clade, but with a trichotomy, supported by the characters ‘47, state 1’ and ‘48, state 1’ that concern the particular shape of the pterostigmata. The polytomy is due to the absence of data on the character ‘56’ (cerci) for *Hypoperla*.

Using a Bayesian analysis, we obtained 75,000 cladograms, with a topology of the 50% consensus (see Additional file 4) very close to that of the MP strict consensus cladogram. The unique difference is *Hypoperla* as sister group of crown Acercaria and (*Idelopsocus* + *Palaeomantis*) as sister group of (*Hypoperla* + crown Acercaria). This second analysis shows that the relative positions of the Miomoptera and Hypoperlidae are uncertain. The Miomoptera are likely to be the sister group of the Hypoperlidae, or the sister group of the (Hypoperlidae + crown Acercaria), or even the two groups are paraphyletic.

Nevertheless, the difference in the structure of the cerci (one-segmented vs. pluri-segmented) between the Hypoperlidae and the Miomoptera is sufficient to distinguish the two groups at this stage of knowledge. Thus we keep the two orders Miomoptera and Hypoperlida separately.

Whether they can still be considered as separate orders is another question that will need further discoveries of more complete specimens to be solved. The problem is similar to the situation between the Timemodea and the Euphasmatodea in Phasmatodea [72].

## Discussion

### Limits of the Hypoperlida

As the Hypoperlidae, type family of the Hypoperlida, are Acercaria, we have to determine if the other taxa currently considered as Hypoperlida are also related to the Acercaria.

Shcherbakov [57] divided the Hypoperlida into the suborders Hypoperlinea [‘ancestral to Psocida (and other Acercaria)’, with the two families Hypoperlidae Martynov, 1928 [1] and Ampelipteridae Haupt, 1941 [73], and Strephocladinea. Later, Rasnitsyn [1] considered this group to consist of three suborders: Hypoperlina (viz. Hypoperlidae Martynov, 1928 [1], Ampelipteridae Haupt, 1941 [73], see below), Strephocladina Martynov, 1938 [74], and Perielytrina Zallesky, 1948 [60] (*Perielytron* Zallesky, 1948 [60]).

Recently, Emeljanov [75] stated ‘When considering the characteristic and description of the order Hypoperlida’ in the Historical Development of the Class Insecta (1980) [76] and the History of Insects (2002) [1], one gets the impression that this order includes two different groups: Hypoperlida proper (= Hypoperlina) and Strephocladina; the latter should instead be treated as an order within Dictyoneuridea’ (= ‘Pseudorhynchota’ sensu Emeljanov [75], also = Palaeodictyopteroidea). Note that the name Pseudorhynchota was preoccupied by the Pseudorhynchota Cholodkovsky 1903 [77], a junior synonym of the Anoplura Leach, 1815 [78].

Remark. Rasnitsyn [2: p. 115] indicated that the three Palaeozoic genera *Rhipidioptera* Brongniart, 1893 [79], *Psoroptera* Carpenter, 1976 [80], and *Homoeodictyon* Martynov, 1937 [81] ‘may belong to Hypoperlida, though their position there is tentative at best’. Aristov [82] placed *Psoroptera* and the Psoropteridae Carpenter, 1976 [80] in the Cnemidolestida. *Homoeodictyon* has a dense venation with archaedictyon and the basal part of wing missing, so that it is certainly not related to the Acercaria [81]. Hörnschemeyer and Stapf [83] placed *Rhipidioptera* in the Blattinopsidae, a family not related to the Hypoperlidae or the Acercaria at all [84].

#### Problem of the Perielytrina

*Perielytron mirabile* Zalessky, 1948 [60] is an enigmatic Permian insect having sclerotized forewing with poorly known venation, and lacking synapomorphies with Acercaria [1, 60]. We consider it as a ‘Neoptera *incertae sedis*’.

#### Problem of the Strephocladina

Strephocladina *sensu* Rasnitsyn [1] comprise the Palaeozoic families Synomaloptilidae Martynov, 1938 [74], Tococladidae Carpenter, 1966 [82, 85, 86, 87], Heteroptilidae Carpenter, 1976 [80], Nugonioneuridae Carpenter, 1976 [80], and Anthracoptilidae Handlirsch, 1922 [88], the Strephocladidae being a junior synonym of this last family [89]. The Tococladidae are Archaeorthoptera [90], a clade with a wing venation different from those of the Acercaria (see above). Anthracoptilidae belongs to Paoliida [89], a clade related to Dictyoptera. Thus, the group Strephocladina is polyphyletic and should be abandoned as currently defined.

Anthracoptilidae, Heteroptilidae, and Nugonioneuridae have CuA emerging from a common stem with CuP [80]. This character excludes them from having any closer affinities with pan Acercaria and Hypoperlidae (see diagnosis of the Acercaria).

Thus, the wing venation of all these ‘Strephocladina’ greatly differs from the acercarian type.

The family Anthracoptilidae (= ‘Strephocladidae’) is not related to the Palaeodictyoptera, refuting Emeljanov [75]’s hypothesis of a link between Strephocladina and ‘Dictyoneuridea’.

The Synomaloptilidae currently comprise the three genera *Synomaloptila* Martynov, 1938 [73], *Rhinomaloptila* Rasnitsyn, 1977 [43], and *Mycteroptila* Rasnitsyn, 1977 [43]. Carpenter [32] included *Synomaloptila* in the Caloneurodea but Béthoux et al. [91] excluded this taxon from this group. The wing venations of *Rhinomaloptila* and *Mycteroptila* are very incompletely known, and do not allow a correct placement of these genera [43, 92]. *Synomaloptila* should be revised before its placement.

Some ‘Strephocladina’ (e.g. *Synomaloptila*) have an elongate head with apparently beak-like mouthparts. Such superficial similarities do not support a phylogenetic relationship of these insects and Acercaria. As modern Psocodea lack such elongate mouthparts, this character is clearly not a ground plan autapomorphy of Acercaria. Also, Palaeodictyoptera have elongate sucking-piercing mouthparts, which is clearly a convergent character with Hemiptera (see below).

#### Problem of the Hypoperlina

Shcherbakov [56] divided the Hypoperlina (his Hypoperlida) into two Palaeozoic families, Hypoperlidae and Ampelipteridae (a family he synonymized with the Protoprosbolidae Laurentiaux, 1952 [93] and the Fatjanopteridae Martynova, 1961 [63]. The same author also listed within Ampelipteridae the genera *Tshunicola* Rasnitsyn, 1977 [43] and *Tshekardobia* Rasnitsyn, 1977 [43] [Rasnitsyn [2: fig 120] confirmed these placements but included the latter genus in Hypoperlidae. Rasnitsyn [1] added the Carboniferous genera *Limburgina* Laurentiaux, 1950 [94], *Aenigmatodes* Handlirsch, 1906 [21], *Gyrophlebia* Handlirsch, 1906 [21], *Protopachytylopsis* Laurentiaux and Laurentiaux-Vieira, 1981 [95], *Anthraconeura* Laurentiaux and Laurentiaux-Vieira, 1980 [96], and, preliminarily, *Mixotermes* Sterzel, 1881 [97], *Pruvostia* Bolton, 1921 [98], and *Boltonocosta* Carpenter, 1986 [99] in Hypoperlina.

The Protoprosbolidae were recently revised, restored, and re-transferred to Hemiptera [5, 100]. *Ampeliptera limburgica* Pruvost, 1912 [101], type species of Ampelipteridae, has been placed in Archaeorthoptera [12, 73]. *Fatjanoptera mnemonica* Martynova, 1961 [63], type of Fatjanopteridae, is an enigmatic taxon strongly differing from *Ampeliptera*. Unlike *Ampeliptera*, *Fajanoptera* possesses a net of cells between the main wing-veins, and, more notably, *Fatjanoptera* has a convex CuA and a concave CuP, emerging from a common stem (see Huang et al. [3]: Figs S9B, S9E), dissimilar to venation in acercarian orders and Archaeorthoptera. *Fatjanoptera* also possesses at least three anal veins, unlike Acercaria and Hypoperlidae. Fatjanopteridae should be restored as a separate family, and placed outside of Archaeorthoptera, Acercaria, and ‘Hypoperlida’ (see below for venation of the Hypoperlidae). *Fatjanoptera* was originally considered to be in Holometabola, related to Raphidioptera [63]. Some aspects of the wing venation in *Fatjanoptera* are reminiscent to those of Holometabola in the distal fusion of ScP with RA and the presence of a pterostigmal-like zone, defined by small veinlets between RA and the costa. Nonetheless, placement of *Fatjanoptera* will remain uncertain until discovery of fossils preserving its body structures.

Béthoux and Nel [12] placed *Protopachytylopsis* in Panorthoptera, unrelated to *Ampeliptera*. Béthoux [102] revised *Anthraconeura,* transferring it to Archaeorthoptera. The description of *Limburgina* was based on the distal two-thirds of its fore (?) wing. However, the bases of the cubital veins are not preserved [94] in its type specimen, thus preventing the distinction between archaeorthopteran or acercarian venation patterns. We consider *Limburgina* as Neoptera *incertae sedis*.

The basal portions of the wings (especially the bases of CuA and CuP) of *Boltonocosta splendens* Bolton, 1912 [103]), *Mixotermes lugauensis* Sterzel, 1881 [97], and *Aenigmatodes danielsi* Handlirsch, 1906, are not well preserved in their respective type specimens [21, 97, 103]. Thus, the taxonomic affinities of these fossils cannot be firmly established. Nevertheless, available wing venation patterns of these specimens lack any of the synapomorphies to place them in Acercaria. Béthoux [102] indicated that the type specimen of *Gyrophlebia longicollis* Handlirsch, 1906 is poorly preserved and its taxonomic placement cannot be correctly determined. *Pruvostia spectabilis* Bolton, 1921 has a venation typical of Anthracoptilidae [98], with a convex CuA having three, clearly concave anterior branches, and CuA and CuP having a common stem. *Tshunicola carbonarius* and the five species of *Tshekardobia* have a wing venation similar to Acercaria in having a reduced number of branches of main veins and few crossveins. But, the organization of the cubital veins in these taxa requires reexamination.

Novokshonov [104] and Rasnitsyn [2] added the Permian Asiuropidae Novokshonov, 1997 [104] (single genus *Asiuropa* Novokshonov, 1997 [104]) to Hypoperlida. *Asiuropa uralensis* Novokshonov, 1997 [104] superficially resembles some Acercaria by having few crossveins between RA and RP and other main veins. However, *A. uralensis* differs from *Hypoperla* and other acercarian-like insects by possessing numerous branches of CuA. The organization of the basal parts of veins R, M, CuA, and CuP is not known. A revision of the type material will be necessary to determine if its venation is of acercarian type.

Rasnitsyn [2] added the Permian Letopalopteridae Martynova, 1961 (with two genera *Letopaloptera* Martynova, 1961 [63] and *Permindigena* Novokshonov, 1998 to Hypoperlina [63, 92]. Novokshonov and Willmann [105] revised *Letopaloptera* and retained it in Hypoperlida. But, Aristov and Rasnitsyn [106] synonymized Letopalopteridae with Permembiidae Tillyard, 1937 [107] and transferred this family into the extinct order Miomoptera (see above). The wing venation of these insects greatly differs from those of Hypoperlidae and Acercaria, most importantly in the presence of a common stem of CuA with CuP.

Rasnitsyn and Aristov [in 47] placed the Ischnoneuridae (and the genus *Ischnoneura* Brongniart, 1893 [79]) in the Hypoperlina. Béthoux and Nel [108] considered that this taxon belongs to the Archaeorthoptera, confirmed in [89].

All of the aforementioned fossils, previously considered as Hypoperlina, are not closely related to Acercaria or to Hypoperlidae (see below). Therefore, we exclude them from Hypoperlida. We consider that the Hypoperlida are reduced to the sole Hypoperlidae.

Remark. The enigmatic Permian fossils *Sojanopus festivum* Novokshonov, 2002 [59] (unique representative of the family Sojanoperidae Novokshonov, 2002 [59]) and *Montanuralia aeria* Novokshonov, 1998 [54] (unique representative of the family Montanuraliidae Novokshonov, 1998 [92]), have been considered as possible Hypoperlidea [59, 92]). They share with Acercaria the CuA basally fused with R + M and re-emerging distally, with a cua-cup between it and the concave CuP. But their wing venations are highly simplified without other crossveins. *Montanuralia aeria* has five tarsomeres, suggesting a very basal position in this clade. Their exact relationships with the other Acercaria remain uncertain.

Aristov [87] considered the monospecific family Permetatoridae Novokshonov, 1999 as belonging to the Hypoperlida. He erroneously attributed this position to Novokshonov [86] who considered this family as ‘Ordinis incertis’. The wings of *Permetator semitritus* are incompletely preserved. Novokshonov [86] considered that this taxon had a common stem Cu of CuA and CuP and a ‘M5’ between M and CuA, but he did not precise the convexity of these different veins, so that only a revision of the type material would allow to determine if his ‘M5’ and his ‘CuA’ are convex or concave, which would completely change the position of this taxon. Nevertheless, the presence of numerous crossveins in all wings, and especially in the area between C and ScP put serious doubts on an attribution close to the Hypoperlidae and the Acercaria.

### Relationships between Miomoptera, Acercaria, and Palaeodictyopterida

The ‘Hypoperlida’ sensu Rasnitsyn [2] is considered as paraphyletic group giving rise to ‘Dictyoneuridea, Psocidea and Cimicidea’ (respectively palaeodictyopteridan and acercarian orders). The proposed ‘clade’ (Hypoperlida + Palaeodictyopterida + Acercaria) is allegedly supported by the presence of ‘maxilla with lacinia rod- or stylet- like’. This character, in most cases, is not visible in compression fossils and also difficult to discern in amber material. Even it is absent in the Hypoperlidae.

The Palaeodictyopterida have either been considered as member of Palaeoptera or as sister group of Neoptera (Sroka et al. [109], but certainly do not nest within Neoptera, while Acercaria is a subgroup of crown group Neoptera. The fact that Palaeodictyopterida and Acercaria are not closely related is strongly supported by morphological analyses [110].

Rasnitsyn [2] considered the piercing rostrum of Palaeodictyoptera and Hemiptera as homologous and derived from a hypoperlidan ancestor. Kukalová-Peck [111] presented a detailed reconstruction of palaeodictyopteroid mouthparts, with structures (lacinia, ante- and postclypeus, mandibular condyles, etc.) generally unavailable for observation in fossils, or undissected modern insects. Other interpretations by Kukalová-Peck [112], Laurentiaux [93], or even Dohrn [113], remain more reasonable, describing very long stylet-like mandibles, and long maxillary palps, but without information on other parts such as laciniae. Prokop et al. [114] demonstrated these parts with microstructures by the use of ESEM on *Brodioptera sinensis* (Megasecoptera) bearing presumably shorter labium that consists of a pair of lobes. Even though these structures are reminiscent of those of Hemiptera (except presence of maxillary palps), they are certainly the result of convergence as already proposed by Laurentiaux [64] and Emeljanov [115], and are not synapomorphies with those Acercaria with piercing mouthparts. All other structures (especially the wing venation) exhibit no synapomorphies between Palaeodictyopterida and Acercaria.

The wing venations of Hypoperlidae and Palaeomanteidae lack any synapomorphy with the palaeodictyopteridan groups (Dictyoneuridea *sensu* Rasnitsyn [2]). In particular the common stem R + M + CuA, present in the Hypoperlidae and the Acercaria, is absent in palaeodictyopteridan orders. Also, Hypoperlidae has only two convex simple anal veins, identical to Acercaria, but different from the anal veins of Palaeodictyoptera, where there are numerous anal veins reinforced by a prominent anal ridge (the so-called ‘anal brace’). These neopteran families cannot be considered as members of a grade that would have given rise to any palaeopterous insects.

Rasnitsyn [2] considered the mouthparts as diagnostic characters for the order Hypoperlida. He described them as ‘chewing though often beak-like elongate, with lacinia rod- or styletlike, clypeus convex indicating strong cibarial muscles, or, if flat, mandibles and laciniae long, jointly forming short beak’. Such structures are barely visible in the few described Hypoperlidae with preserved bodies. In fact, the mouthparts of *Idelopsocus splendens* (specimens PIN 1700/3298 and PU 2/129), *Idelopsocus diradiatus*, and *Idelopsocus galinae* are not particularly elongate and resemble the mouthparts of Psocodea, especially in the entire gena [56, 61].

## Conclusions

The direct re-exam of the type material of *Hypoperla* and *Palaeomantis*, and the study of new specimens allows us to restrict the limits of the two orders Hypoperlida and Miomoptera to the sole type families, to consider them as Acercaria sensu lato on the basis of clearly defined synapomorphies, and to exclude all the other families previously included in these orders. We also exclude any affinities between the Hypoperlida and the Palaeodictyopterida, and affinities between the Miomoptera sensu stricto and the Holometabola.

## Methods

### Wing venation terminology and abbreviations

Venation nomenclature is following the concept of Nel et al. (2012) [5]. Wing venation abbreviations: (ScP—subcosta posterior, RA/RP—radius anterior/posterior, M—indistinguishable polarity of median vein, CuA/CuP—cubitus anterior/posterior, A1/A2—first/second anal vein; pt–pterostigma; cf–claval furrow; cua-cup–specialized crossvein proximally concave and distally convex between CuP and CuA).

### Line drawings and photographs

The venation patterns were drawn directly using a camera lucida. Photographs were taken with digital camera Canon D550 with reverse lens MP-E 65 mm. Original photographs were processed using the image-editing software Adobe Photoshop CS4, and for some images were processed by the focus-stacking software Helicon Focus Pro. Scanning electron micrographs of *Delopsocus latus* were taken by an environmental electron microscope Hitachi S-3700 N in the National Museum in Prague.

### Phylogenetic analysis

The phylogenetic analysis was performed using the software Win-Paup4b10 [116], Bandb option. The matrix was established using Mesquite 3.03 [117]. The characters are equally weighted and unordered. The chosen outgroups are: *Periplaneta* sp. (Dictyopterera), *Eusthenia* sp. (Plecoptera) and *Xyela* sp. (Holometabola: Hymenoptera).

The Bayesien analyses were conducted using the Mk model of discrete character evolution, as suggested by Wright and Hillis [118], with MrBayes 3.1.2 [119]. We ran the analyses for 50 millions of generations with the command stoprule = yes, sampling tree every 500 generations. We used Tracer 1.5 [120] to check that our effective sample size was large enough for a meaningful estimation of parameters and to assess the burn-in. Finally, we checked for convergence of our results ensuring that the potential scale-reduction factor approached 1.0 for all parameters.

## Additional files

Additional file 1: Phylogenetic analysis and list of characters. (DOC 81 kb)

Additional file 2: Coding of characters and list of taxa used in this study. (XLS 100 kb)

Additional file 3: Nexus file used for the phylogenetic analysis. (NEX 9 kb)

Additional file 4: Bayesian 50% consensus tree. (TIFF 788 kb)
