# Supplementary material for: Redefining the extinct orders Miomoptera and Hypoperlida as stem acercarian insects
Source: BMC Evol Biol. 2017 Aug 25;17:205. doi: 10.1186/s12862-017-1039-3 (PMC5574135; doi:10.1186/s12862-017-1039-3)
Supplement: Supplementary file 1 — Phylogenetic analysis and list of characters. (DOC 81 kb) [file 12862_2017_1039_MOESM1_ESM.doc]

**List of taxa used in the phylogenetic analysis**

Outgroups:

Blattodea:

*Periplaneta americana* (Linnaeus, 1758) (extant)

Plecoptera:

*Eusthenia costalis* Banks, 1913 (extant)

Hymenoptera:

*Xyela julii* (Brébisson, 1818) (extant)

Ingroups:

Hypoperlida:

*Hypoperla elegans* Martynov, 1928 (based on reexamined photographs of the type wings) (Permian)

*Idelopsocus splendens* (Zalessky, 1948) (based on revision of specimens PIN 1700/3298 and PU 2/129) (Permian)

Miomoptera:

*Palaeomantis aestiva* Novokshonov, 2000 (based on reexamined holotype PIN No. 1700-4093 and paratype PIN No. 1700-1381 (Early Permian)

Permopsocida:

*Archipsylla sinica* Huang et al, 2008 (Middle Jurassic)

*Dichentomum tinctum* Tillyard, 1926 (based on present revision) (Permian)

*Permopsocus latipennis* Tillyard, 1926 (Permian)

*Psocorrhyncha burmitica* gen. nov., sp. nov. (Cretaceous)

Psocodea:

*Burmacompsocus perreaui* Nel & Waller, 2007 (Compsocidae) (Cretaceous)

*Libanomphientomum nudus* Choufani et al., 2011 (Amphientomidae) (Cretaceous)

Thripida:

*Moundthrips beatificus* Nel et al., 2007 (Cretaceous)

*Thrips tabaci* Lindeman, 1889 (extant)

Hemiptera:

*Archescytina* sp. (Archescytinidae, supposed most basal clade of Hemiptera, specimen with body preserved)

*Southia opposita* (F., 1803) (Fulgoromorpha: Kinnaridae) (extant)

**Characters and character states used in the phylogenetic analysis**

1. Head: (0) not opisthognathous; (1) opisthognathous, orientated obliquely, with mouthparts pointed backward (Palaeozoic and some Mesozoic Thripida have a prognathous or hypognathous head, while the head is opisthognathous in modern Thysanoptera [1]. The opisthognathy cannot be considered a synapomorphy of Thripida and Hemiptera. The Permopsocida have hypognathous heads) (state 0 for *Psocorrhyncha*)
2. Sclerotized ring at base of first antennal flagellomere, inside pedicel: (0) absent; (1) present (a character of Hemiptera and modern Thysanoptera [2,3]) (state 0 for *Psocorrhyncha*)
3. Rupturing mechanism at base of antennal flagellum: (0) absent; (1) present (a character of Psocodea [3,4]) (state 0 for *Psocorrhyncha*)
4. Flagellomeres annulated with cuticular sculpture: (0) present; (1) absent (annulation is present in Psocodea: Troctomorpha, in some Thripida, Hemiptera: Aphidoidea, Isoptera, Mantophasmatodea, Ephemeroptera, and Plecoptera) (state 0 for *Psocorrhyncha*)
5. Insertion of scape on head capsule by a dicondylar articulation (acute lateral antennifer and weaker median articulation point on head capsule): (0) present; (1) absent (a dicondylar articulation occurs in modern Thysanoptera [3], Orthoptera, Phasmatodea, and Thysanura [5-7], while other insects have a ball-and-socket joint [7]. Psocodea have a single condyle or no condyle [3,8]. While Heming [3] considered the dicondylar articulation as derived in Thysanoptera, its presence in Thysanura, Orthoptera, and Phasmatodea suggests it could be a plesiomorphy for the Insecta) (state 0 for *Psocorrhyncha*)
6. Position of anterior tentorial pits: (0) frontal side of head; (1) shifted dorsally (the anterior tentorial pits are absent in Anoplura and Rhynchophthirina, not considered here; they are shifted dorsally in Hemiptera and modern Thysanoptera, but not in Palaeozoic and Mesozoic Thripida [4,9]) (state 0 for *Psocorrhyncha*)
7. Presence of a sub-horizontal corypha and a subvertical anterior metopa bearing the ocelli: (0) no (Psocodea, Thripida); (1) yes (state ‘0’ occurs in outgroups, Psocodea and Thripida, state ‘1’ occurs in Hemiptera: Fulgoromorpha [9-11]) (state 1 for *Psocorrhyncha*)
8. Ocell-ocular distance < inter-ocellular distance: (0) no ; (1) yes (state ‘1’ occurs in those Hemiptera with a broad clypeo-frons [12]) (state 1 for *Psocorrhyncha*)
9. Clypeus divided by a furrow into ante- and postclypeus: (0) no; (1) yes (state ‘1’ in some Hemiptera (e.g. Cicadoidea) but not all (e.g Aphidoidea), Thripida, and some Psocodea [13]) (state 1 for *Psocorrhyncha*)
10. Postclypeus: (0) not very large and bulbous; (1) large, bulbous, with large cibarial dilator muscles (this character state is currently assigned to the Acercaria [13,14]), but the postclypeus is not as large and bulbous in the Palaeozoic or Mesozoic Thripida nec. Thysanoptera as in Psocodea, extant Thysanoptera, and Hemiptera. Therefore the large postclypeus of Psocodea, extant Thysanoptera and Hemiptera is certainly a convergence. In Psocodea, the frons is well separated from the postclypeus, unlike in modern Thysanoptera, and probably Hemiptera, although terminology for the latter clade is controversial [10,13,15]) (state 0 for *Psocorrhyncha*)
11. Paraclypeal lobes: (0) not separated and not distinct from median part of (ante)-clypeus; (1) separated and distinct from median part of (ante)-clypeus (Presence of two relatively sclerotized paraclypeal lobes [16] is an apomorphic character present in recent and fossil Thripida^58^. Hemiptera also have sclerotized sclerites in the same position as the paraclypeal lobes of Thripida and of *Psocorrhyncha*. Some authors confused the mandibular plate (lora) for paraclypeus (see [10] for summary of diverse opinions). Spangenberg et al. [16] and Spangenberg [17] confirmed the opinion of Singh [18] about the fact that the paraclypeus of Hemiptera is a structure different from the mandibular plate. In Coleorrhyncha [16], the paraclypeal lobes are visible in dorsal view, placed laterally to the anterior part of the anteclypeus while mandibular plates are visible only in lateral view; these structures are fused but separated internally by a ‘distinct cresent-shaped apodeme’[16]. In the aphidoidean *Stomaphis*, the paraclypeal lobes are very broad structures. The clypeus of the Psocodea, Hypoperlidae, and other Insecta is not clearly differentiated into paraclypeal lobes and a median part. This character constitutes a potential synapomorphy of a clade comprising Permopsocida, Thripida, and Hemiptera. *Stenopsocidium elongatum* could also have two sclerotized paraclypeal lobes. Also *Dichentomum tinctum* has two small rounded sclerites at the base of the labrum corresponding to paraclypeal lobes, (state 1 for *Psocorrhyncha*)
12. Median part of (ante)-clypeus: (0) not membraneous; (1) membraneous (state ‘1’ in Thripida [9]; a potential synapomorphy of Permopsocida and Thripida, modified in Hemiptera in relation to the hyper-development of the clypeus) (state 1 for *Psocorrhyncha*)
13. Labrum: (0) not elongate, less than two times longer than broad; (1) elongate, two times longer than broad or more (state ‘1’ in Hemiptera and Thripida) (state 1 for *Psocorrhyncha*)
14. Left mandible: (0) not stylet-like; (1) stylet-like (state ‘1’ in Hemiptera and Thripida) (state 0 for *Psocorrhyncha*)
15. Right mandible: (0) not stylet-like; (1) stylet-like (state ‘1’ in Hemiptera; the elongate mandibles with a broad base together with the elongate labrum in *Psocorrhyncha* fits well with the “Hypothetical scheme of transformations of chewing mandibles into stylets” proposed by Emeljanov [19], placing *Psocorrhyncha* between his steps “(1)” (psocodean state) and “(2)”. Nevertheless the mandibles of Permopsocida are clearly plesiomorphic compared to the stylet-like mandibles of Thripida and Hemiptera) (state 0 for *Psocorrhyncha*)
16. Right mandible: (0) present; (1) absent, mouthcone asymmetrical (state ‘1’ in Thripida [1]) (state 0 for *Psocorrhyncha*)
17. Maxillary lacinia: (0) in direct contact with stipes; (1) not in direct contact with stipes, probably independently movable (putative apomorphy of Acercaria [4], there is an intermediate structure between the lacinia and the stipes in modern Thysanoptera) (state 1 for *Psocorrhyncha*)
18. Lacinia: (0) with at least one subapical tooth; (1) without any subapical tooth (in Orthoptera, Phasmatodea, Plecoptera, and Psocodea, the lacinia has at least one strong subapical tooth, except in few Caeciliidae, while in Thripida and Hemiptera there is only an acute apical tooth) (state 1 for *Psocorrhyncha*)
19. Lacinia: (0) distally broad; (1) stylet-like distally (a broadened distal part of lacinia is a plesiomorphic character state present in Psocodea, compared to the acute and thin lacinia of Thripida and Hemiptera [8]; note the eucinetid beetle *Jentozkus plaumanni* has stylet-like lacinia, plus galea) (state 0 for *Psocorrhyncha*)
20. Lacinia: (0) not elongate; (1) elongate (state ‘1’ in Acercaria, but elongate lacinia cannot be considered as a strict synapomorphy of Acercaria because elongate lacinia occur frequently when the head and mouthparts are elongate (e.g., the mecopteran genus *Panorpodes*) (state 1 for *Psocorrhyncha*)
21. Cardo and stipes: (0) separated by a furrow; (1) fused (The cardo and stipes separated by a furrow is a plesiomorphy relative to their fusion in Psocodea [4,14]) (state 0 for *Psocorrhyncha*)
22. Gena: (0) not subdivided into two parts, (1) subdivided into two parts by a strong furrow (The gena is subdivided into two parts by a strong furrow in *Psocorrhyncha*. Such a subdivision of the gena is absent in Psocodea and the Hypoperlidae, but visible in *Dichentomum*. There is a controversy about the origin of the maxillary lobe of Hemiptera of genal origin [20,21], versus of appendicular origin (maxilla) [15,17]. Duporte [22] proposed that the maxillary plate could be of composite origin, due to the fusion of cardo and stipes, and that both latter in turn are fused with the genae and postgenae. Presence of a posterior lobe of gena in *Psocorrhyncha* would support the hypothesis of Bourgoin [20] because this taxon has a ‘normal’ maxilla not fused with the gena, and in many Hemiptera there is continuity without any maxillary suture between the posterior part of the gena and the maxillary plate. Nevertheless the problem will be really solved using the tools of the genetic of the development. The anterior part of the gena is currently called lora (for non-heteropteran Hemiptera), or mandibular plates (for Heteroptera) [16,20]. The mid Jurassic Permopsocida appear to also have a subdivision of the gena. The Thripida have also a gena subdivided into a long mandibular plate in lateral position in front of the base of the antenna plus a posterior part below the eye (visible in the Cretaceous thripidan *Moundthrips*, and present in the early nymphs of modern *Heliothrips* or Haplothrips [23,24]. A genal fissure is also present in the modern Tubulifera [25]. The anterior mandibular plate closes the mouthcone laterally in *Moundthrips*, but it appears fused with the maxilla in modern Thysanoptera. Emeljanov [19] proposed hypothetical stages of transformation from the ‘psocodean’ head to the ‘hemipteran’ one, with two structures progressively appearing and developing and corresponding to the mandibular and maybe the maxillary lobes, but he misplaced these structures anteriorly to the gena) (state 1 for *Psocorrhyncha*)
23. Maxillary palp: (0) five-segmented; (1) four-segmented, (2) less than four-segmented (Hemiptera and Thripida have state ‘2’) (state 1 for *Psocorrhyncha*)
24. Last maxillary palpomere: (0) inserted normally on penultimate; (1) inserted apically on penultimate, penultimate cut obliquely at its apex (*Psocorrhyncha* unique apomorphy)
25. Last maxillary palpomere: (0) without broad flat sensillar zone; (1) with broad flat sensillar zone (*Psocorrhyncha* unique apomorphy, unknown in other Permopsocida)
26. Mentum: (0) not elongated; (1) elongated (state ‘0’ in Psocodea and Hypoperlidae, the mentum are not elongate; state ‘1’ in Hemiptera and Thripida [26] (state 1 for *Psocorrhyncha*)
27. Labial palps: (0) with more than two segments; (1) absent or strongly reduced (Presence of three-segmented labial palps in *Psocorrhyncha* is plesiomorphic. Psocodea and Thripida have labial palps one- or two-segmented, while they are lost in Hemiptera) (state 0 for *Psocorrhyncha*)
28. Hypopharynx: (0) not expanded posteriorly; (1) expanded posteriorly (state ‘0’ in Psocodea, state ‘1’ in Hemiptera, modern Thysanoptera, and at least in *Moundthrips* among Mesozoic Thripida [19,27]) (state unknown for *Psocorrhyncha*)
29. Cibarial water-vapour uptake apparatus: (0) absent; (1) present (state ‘1’ in Psocodea [4]) (state unknown for *Psocorrhyncha*)
30. Pearman's organ on hind coxa: (0) absent; (1) present (state ‘1’ Psocoptera excl. Liposcelidae) (state unknown for *Psocorrhyncha*)
31. Number of tarsomeres (multistate): (0) five; (1) four; (2) three or less (five-segmented tarsi in the ground plan of Pterygota; three-segmented tarsi in the ground plan of Plecoptera, state ‘2’ in Zoraptera, Psocodea, Thripida, and Hemiptera; five-segmented tarsi in Hypoperlidae, four-segmented tarsi in Permopsocida; it is likely that reduction in the number of tarsomeres occurred convergently in Zoraptera, Psocodea, and the clade Thripida + Hemiptera) (state 1 for *Psocorrhyncha*)
32. Paired tarsal plantulae: (0) present; (1) absent (state ‘1’ is a character of Eumetabola = Acercaria + Holometabola; Beutel & Gorb [28] indicated the presence of ‘euplantulae’ in some Mallophaga, but these are unpaired structure [29], probably non homologous [30] to the euplantulae of the polyneoptera) (state 1 for *Psocorrhyncha*)
33. Claws: (0) not reduced in adult; (1) reduced in adult (state ‘1’ in fossil and modern Thripida) (state 0 for *Psocorrhyncha*)
34. Arolium: (0) broad and fleshy; (1) arolium broad but retractile; (2) arolium reduced, only a pulvillus inserted at base of claw (state ‘0’ in *Psocorrhyncha*, Xylelidae, many Polyneoptera, Hemiptera; state ‘1’ in Thripida [9]; state ‘2’ in Psocodea [4])
35. In wing articulation, humeral plate (HP) and basisubcostale (BSc): (0) separated; (1) united (state ‘1’ apomorphy of Acercaria [31]) (state 1 for *Psocorrhyncha*)
36. In wing articulation, BSc and second axillary sclerite (2Ax): (0) separated; (1) fused (state ‘1’ in Hemiptera [31]; state unknown in Thripida) (state 0 for *Psocorrhyncha*)
37. Fringe on posterior edge of wing: (0) absent; (1) present. (state ‘1’ in Thripida^7^) (state 0 for *Psocorrhyncha*)
38. Forewings: (0) not more sclerotized than hind wings; (1) at least slightly more sclerotized than hind wings (state ‘1’ in some modern Hemiptera [32]) (state 0 for *Psocorrhyncha*)
39. Wings: (0) hind wings not much smaller than forewings; (1) hind wings much smaller than forewings (state ‘1’ in Psocodea but also in Hemiptera: Aphidoidea [32]) (state 0 for *Psocorrhyncha*)
40. A common stem R+M+CuA: (0) absent; (1) present (state ‘1’ convergently present in Archaeorthoptera and Acercaria [33,34] (state 1 for *Psocorrhyncha*)
41. M (plus CuA if fused basally with radius) separates from R: (0) well distal of wing base; (1) very close to wing base (state ‘1’ is proper to the Psocodea, fossil and modern, except some Troctomorpha [33]) (state 0 for *Psocorrhyncha*)
42. A neutral crossvein cua-cup between concave CuP and convex CuA, weaker than CuA: (0) absent; (1) present (state ‘1’ in Acercaria^7^) (state 1 for *Psocorrhyncha*)
43. Radial stem at point of re-emergence of CuA and M: (0) not displaying a pronounced posterior angle; (1) displaying a strong posterior angle (Such an angle appears to be present in the hemipteran ground plan, as it can be observed in Archescytinidae and many Fulgoromorpha, but not in Psocodea or Thripida) (state 1 for *Psocorrhyncha*)
44. Areola postica: (0) absent; (1) present, longer than high; (2) present, higher than long (States ‘1’ or ‘2’ in Acercaria, CuA-fork is reduced in Thripida and few Psocodea) (state 2 for *Psocorrhyncha*)
45. Vein M: (0) forked into many branches; (1) forked twice into four branches M1-M2 and M3-M4; (2) forked into three pectinate branches (hemipteran ground plan?); (3) only forked once into two branches or less (State ‘1’ in Permopsocida; the three states ‘1’, ‘2’, and ‘3’ are present among various taxa in Psocodea and Hemiptera; state ‘3’ in Thripida)
46. RP: (0) forked; (1) unforked (State ‘1’ in the majority of Hemiptera, but not all) (state 0 for *Psocorrhyncha*)
47. Pterostigma in forewing: (0) absent; (1) present but not limited by costal wing margin and vein RA, more sclerotized than rest of wing; (2) present, limited by costal wing margin and vein RA, more sclerotized than rest of wing (State ‘2’ in psocodean ground plan; Thripida have no pterostigmata; Hemiptera have forewing pterostigmata in their ground plan, present in Archescytinidae and some Fulgoromorpha, Aphididae, etc.) (state 2 for *Psocorrhyncha*)
48. Pterostigma in hind wing: (0) absent; (1) present but not limited by costal wing margin and vein RA, more sclerotized than rest of wing; (2) limited by costal wing margin and a deep posterior curve of vein RA, more sclerotized than rest of wing (State 2 for Permopsocida, autapomorphy; similar hind wing pterostigmata are also present in holometabolous Raphidioptera; Hemiptera Archescytinidae also have pterostigmata in their fore- and hind wings, but of different shape)
49. Forewing ScP: (0) parallel to radius and fusing with it far from wing base; (1) fused with costa near wing base but re-emerging distally to end in radius; (2) fused with costa near wing base and not re-emerging (homoplastic character states as the two situations ‘0’ and ‘2’ can occur in the same family of Psocodea, and in different taxa of Permopsocida; state ‘1’ occurs also in the psocodean family Lepidopsocidae but with ScP only fused for a short length with costa)
50. Anal veins in fore wings: (0) more than two free anal veins; (1) two free anal veins or less (state ‘1’ in Acercaria) (State 1 for *Psocorrhyncha*)
51. Coupling of fore- and hind wings with stigmapophysis in rest (a blunt chitinous projection at base of pterostigma of forewing): (0) absent; (1) present (State ‘1’ in winged Psocodea) (state 0 for *Psocorrhyncha*)
52. Jugal ‘bar’: (0) absent; (1) present (State ‘1’ in Eumetabola; definitely not present in Zoraptera according to Grimaldi & Engel [14] and Friedemann et al. [4]; contra Wheeler et al. [35]); not discernable in any of the studied fossils)
53. Abdominal sternite 1: (0) present and fully developed; (1) reduced or absent (State ‘0’ in Zoraptera; state ‘1’ in modern Acercaria, except Thysanoptera; Friedemann et al. [4]) (state 1 for *Psocorrhyncha*)
54. Abdominal segment I: (0) not very narrow and reduced; (1) very narrow and reduced (Character state ‘1’ present in all Permopsocida; the hypoperlid *Idelopsocus* has a narrow segment I but less narrow than in Permopsocida; nevertheless some Burmese amber specimens and extant Psocodea (e.g. *Lachesilla*) have a similar constriction, thus this character is subject to homoplasy in Acercaria)
55. Female with reduced abdominal tergites IX and X (thripidan type): (0) no; (1) yes (state ‘1’ in Thripida [9]) (state 0 for *Psocorrhyncha*)
56. Cerci: (0) long and multi-segmented; (1) short and one-segmented; (2) absent (State ‘1’ in Zoraptera; state ‘2’ in Acercaria, except in Hypoperlidae; in Hymenoptera there are ‘cerci’ but it is unclear if they belong to the 10^th^ or the 11^th^ segment [36]) (state 2 for *Psocorrhyncha*)
57. Ovipositor: (0) present and well developed; (1) reduced, of psocodean type (State ‘1’ Psocodea; state ‘0’ in the ground plan of Thripida, a character described by Bourgoin [37]. The female anal appendages of *Psocorrhyncha* are similar to those of Hemiptera: Fulgoromorpha of raking type [37], viz. in the presence of gonapophyses VIII with a raking structure, gonapophyses IX weaker and less sclerotized and broad weakly sclerotized gonoplacs. These anal appendages do not correspond to female anal appendages of thripidan type [9] because *Psocorrhyncha* has reduced tergites IX and X. *Psocorrhyncha* differs from those of the female Psocodea in the strong gonapophyses VIII with raking apparatus [11])
58. Female gonangulum: (0) not fused with tergum IX; (1) fused with tergum IX (State ‘1’ in Acercaria; after Friedemann et al. [4], ‘The gonangulum is fused with tergum IX in Acercaria and Odonata’, and ‘the situation is unknown for Enicocephalomorpha, Dipsocoromorpha, and Phthiraptera’) (state unknown for *Psocorrhyncha*).
59. Gonostyli: (0) present; (1) absent, lost (state ‘1’ in Acercaria, Zoraptera, Embioptera) (state 1 for *Psocorrhyncha*)
60. Male anal appendages more sclerotized, especially with large and strongly sclerotized spoon-like hypandrium: (0) yes; (1) no (State ‘1’ in modern Psocodea [11]. The male anal appendages of *Psocorrhyncha* are more sclerotized than in modern Psocodea, especially in the presence of a large and strongly sclerotized spoon-like hypandrium) (state 0 for *Psocorrhyncha*)
61. Abdominal ganglia: (0) more than two separate ganglia; (1) two separate ganglia; (2) one single ganglionic mass. Two separate abdominal ganglionic complexes are found in Zoraptera. A single ganglionic mass is a possible autapomorphy of Acercaria [11].
62. Lateral hypopharyngeal arm (0) present; (1) absent. The lateral hypopharyngeal arm is absent in Psocodea and Zoraptera. It is present in Thysanoptera, Auchenorrhyncha, Aphidoidea, Psylloidea, Pentatomomorpha, Enicocephalomorpha, Dipsocoromorpha, and Coleorrhyncha. The situation is unknown for Aleyrodidae, and Coccoidea [11].

References

1. Moritz, G. Zur Morphologie und Anatomie des Fransenflüglers *Aeolothrips intermedius* Bagnall. 1. Mitteilung: der Kopf. Zool. Jb. Anat. **107**: 557–608 (1982).

2. Seeger, W. Funktionsmorphologie an Spezialbildungen der Fühlergeissel von Psocoptera und anderen Paraneoptera (Insecta); Psocodea als monophyletische Gruppe. Z. Morphol. Tiere **81**: 137–159 (1975).

3. Heming, B. S. Antennal structure and metamorphosis in *Frankhniella fusca* (Hinds) (Thripidae) and *Haplothrips verbasci* (Osborn) (Phlaeothripidae) (Thysanoptera). Quaest. Entomol. **11**: 25–68 (1975).

4. Friedemann, K., Spangenberg, R., Yoshizawa, K., Beutel, R. G. Evolution of attachment structures in the highly diverse Acercaria (Hexapoda). Cladistics 30: 170–201 (2013). doi: 10.1111/cla.12030.

5. Matsuda, R. Morphology and evolution of the insect head. Mem. Am. Entomol. Inst. **4**: 1–334 (1965).

6. Gewecke, M. Bewegungsmechanismus und Gelenkrezeptoren der Antennen von *Locusta migratoria* L. (Insecta, Orthoptera). Z. Morphol. Tiere **71**: 128–149 (1972).

7. Staudacher, E. M., Gebhardt, M., Durr, V. Antennal movements and mechanoreception: neurobiology of active tactile sensors. Adv. Insect Physiol. **32**:49–205 (2005). doi: 10.1016/S0065-2806(05)32002-9.

8. Badonnel, A. Recherche sur l’anatomie des psoques. Suppl. Bull. Biol. Fr. Belg. 18: 1–241 (1934).

9. Nel, P. *et al*. Redefining the Thripida (Insecta: Paraneoptera). J. Syst. Palaeontol. **12**: 865–878 (2014). doi: 10.1080/14772019.2013.841781.

10. Dmitriev, D. A. Homologies of the head of Membracoidea based on nymphal morphology with notes on other groups of Auchenorrhyncha (Hemiptera). Eur. J. Entomol. 107: 597–613 (2010).

11. Mockford, E. L. North American Psocoptera (Insecta). Flora & Fauna Handbook 10:1–455 (1993).

12. Evans, J. W. The morphology of the head of Homoptera. Pap. Proc. Roy. Soc. Tasm. **1937**: 1–20 (1938).

13. Yoshizawa, K., Saigusa, T. Reinterpretations of clypeus and maxilla in Psocoptera, and their significance in phylogeny of Paraneoptera (Insecta: Neoptera). Acta Zool. **84**: 33–40 (2003). doi: 10.1046/j.1463-6395.2003.00127.x.

14. Grimaldi, D. A., Engel, M. S. Evolution of the Insects. Cambridge: Cambridge Univ. Press (2005).

15. Evans, J. W. The maxillary plate of Homoptera Auchenorrhyncha. J. Entomol. (A) **48**: 43–47 (1973).

16. Spangenberg, R. et al. The cephalic morphology of the Gondwanan key taxon *Hackeriella* (Coleorrhyncha, Hemiptera). Arthropod Struct. Dev. 42: 315–337 (2013). PMID: 23583344.

17. Spangenberg, R. The evolution of head structures in Acercaria (Insecta). PhD Thesis, Friedrich-Schiller-Universität Jena, 413 pp. (2014).

18. Singh, S. Morphology of the head of Homoptera. Res. Bull. Panjab Univ. (NS) **22**: 261–316 (1971).

19. Emeljanov, A. F. Evolutionary scenario of rostrum formation in the Rhynchota. Entomol. Rev. **82**: 1197–1206 (2002).

20. Bourgoin, T. Valeur morphologique de la lame maxillaire chez les Hemiptera; remarques phylogénétiques. Ann. Soc. Entomol. Fr. (NS) **22**: 413–422 (1986).

21. Parsons, M. C. The morphology and possible origin of the Hemipteran loral lobes. Can. J. Zool. **52**: 189–202 (1974).

22. DuPorte, E. M. The anterior tentorial arms in insects and their significance in interpreting the morphology of the cranium of cicadas. Can. J. Zool. **40**: 137–144 (1962).

23. Reyne, A. Untersuchungen über die Mundteile der Thysanopteren. Zool. Jb. Anat. 49: 391–500 (1927).

24. Heming, B. S. Structure and function of the mouthparts in larvae of *Haplothrips verbasci* (Osborn) (Thysanoptera, Tubulifera, Phlaeothripidae). J. Morphol. 156: 1–38 (1978).

25. Bhatti, J. S. New perspectives in the structure and taxonomy of Tubulifera. Zoology **5**: 147–176 (1998).

26. Chaudonneret, J. Les pièces buccales des insectes. Thème et variations. Edition hors série du Bulletin Scientifique de Bourgogne: 1–256 (1992).

27. Nel, P., Azar, D., Nel, A. A new ‘primitive’ family of thrips from the Lower Cretaceous Lebanese amber (Insecta, Thysanoptera). Cretaceous Research **28**: 1033–1038 (2007).

28. Beutel, R. G., Gorb, S. N. Ultrastructure of attachment specializations of hexapods (Arthropoda): evolutionary patterns inferred from revised ordinal phylogeny. Z. Zool. Syst. Evolutionforschung **39**: 177–207 (2001).

29. Kéler, S. Über den feineren Bau der Tarsen bei Pseudomenopon rowanae Keler (Mallophaga). Beitr. Entomol. **2**: 573–582 (1952).

30. Beutel, R. G., Gorb, S. N. A revised interpretation of the evolution of attachment structures in Hexapoda with special emphasis on Mantophasmatodea. Arthropod Systematics & Phylogeny, **64**: 3–25 (2006).

31. Yoshizawa, K., Saigusa, T. Phylogenetic analysis of paraneopteran orders (Insecta: Neoptera) based on forewing base structure, with comments on monophyly of Auchenorrhyncha. Syst. Entomol. **26**: 1–13 (2001). doi: 10.1046/j.1365-3113.2001.00133.x.

32. Hennig, W. Insect phylogeny. Chichester: Wiley and Sons (1981).

33. Nel, A. *et al*. Traits and evolution of wing venation pattern in paraneopteran insects. J. Morphol. **273**: 480–506 (2012). doi: 10.1002/jmor.11036.

34. Béthoux, O., Nel, A. Venation pattern and revision of Orthoptera sensu nov. and sister groups. Phylogeny of Palaeozoic and Mesozoic Orthoptera sensu nov. Zootaxa **96**: 1–88 (2002).

35. Wheeler, W.C., Whiting, M., Wheeler, Q. D., Carpenter, J.M. The phylogeny of the extant hexapod orders. Cladistics **17**: 113–169+404 (erratum) (2001). doi: 10.1006/clad.2000.0147.

36. Dathe, H. H. Hymenoptera. In: Dathe, H. H. editor. Lehrbuch der Speziellen Zoologie, Band 1. Wirbellose Tiere, 5. Teil Insecta. Heidelberg: Spektrum: pp. 585–651 (2003).

37. Bourgoin, T. Female genitalia in Hemiptera Fulgoromorpha, morphological and phylogenetic data. Ann. Soc. Entomol. Fr. (NS) **29**: 225–244 (1993).
