# Supplementary figures and images for: Redefining the extinct orders Miomoptera and Hypoperlida as stem acercarian insects
Source: BMC Evol Biol. 2017 Aug 25;17:205. doi: 10.1186/s12862-017-1039-3 (PMC5574135; doi:10.1186/s12862-017-1039-3)

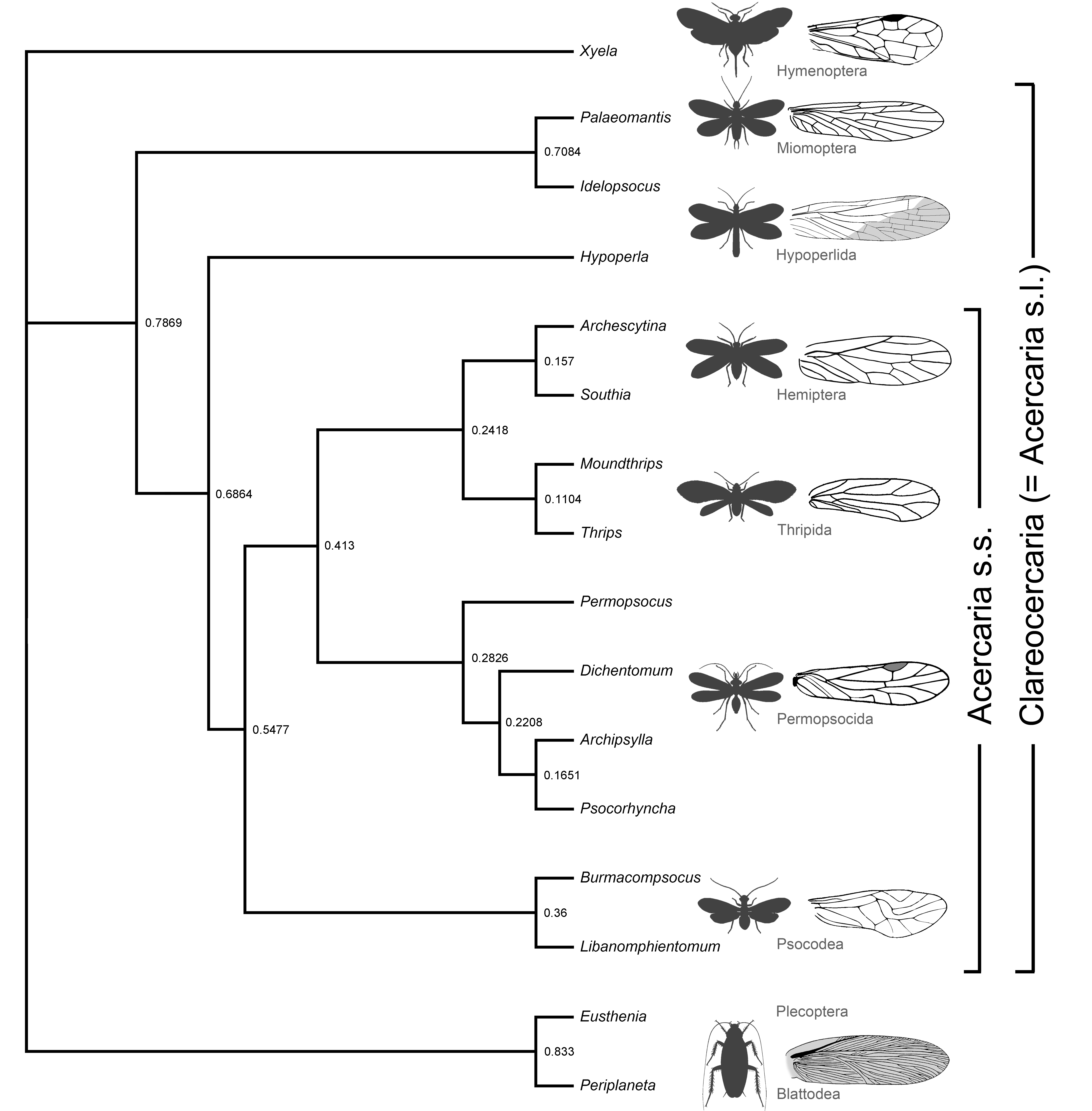

Supplement: Supplementary file 4 — Bayesian 50% consensus tree. (TIFF 788 kb) [file 12862_2017_1039_MOESM4_ESM.tif]
